# Supplementary material for: A Keplerian Ag90 nest of Platonic and Archimedean polyhedra in different symmetry groups
Source: Nat Commun. 2020 Jul 3;11:3316. doi: 10.1038/s41467-020-17198-1 (PMC7335041; doi:10.1038/s41467-020-17198-1)
Supplement: Supplementary file 1 — Supplementary Information [file 41467_2020_17198_MOESM1_ESM.pdf]

Supplementary Information

A Keplerian Ag_{90} Nest of Platonic and Archimedean Polyhedra in Different Symmetry Groups

Su et al

Supplementary Methods.

Materials and reagents

Solvents and reagents (Adamas-Beta®) were purchased from Shanghai Titan Scientific Co., Ltd. Unless otherwise noted, all of the chemicals were reagent grade and used without any further purification. The precursor, [^{t}BuSAg]$_n$, was prepared according to the literature.[1] The solution of $AgNO_3$ (30 mmol, 5.1 g) in 75 mL acetonitrile was mixed with 100 mL ethanol containing ^{t}BuSH (30 mmol, 3.38 mL) and 5 mL Et_3N under stirring for 3 h in the dark at room temperature, then the white powder of [^{t}BuSAg]$_n$ was isolated by vacuum filtration and washed with 10 mL ethanol and 20 mL ether, then dried in the ambient environment (yield: 95 %, based on $AgNO_3$).

Fourier transform infrared spectroscopy (FTIR)

The IR-ATR spectrum was recorded on a PerkinElmer Spectrum Two in the frequency range of 4000–400 cm-1.

Elemental analysis

The elemental analyses (C and H) were determined on a Vario EL III analyzer.

UV/Vis spectroscopy

UV-Vis diffuse reflectance spectra (DRS) were recorded on a Thermo Scientific Evolution 220 UV-visible spectrophotometer equipped with a 60 mm integrating sphere and SPECTRALON® was used as the reference.

Nuclear magnetic resonance (NMR)

The ^{31}P NMR spectrum was recorded in a J. Young NMR tube on Bruker Avance 500 spectrometer. (In general, crystals of **SD/Ag90a** were dissolved in DMSO and digested with excessive 37% HCl. The digestion solution was used directly for ^{31}P NMR measurement.) The chemical shifts are reported in parts per million δ (ppm).

Thermogravimetic analysis (TGA)

Thermogravimetic analysis (TGA) was done in a TA SDT Q600 thermal analyzer at a heating rate of 20 °C min^{-1} under N_2 atmosphere (200 mL min^{-1}) from 20 to 800 °C.

Photoluminescence spectroscopy

Temperature-dependent photoluminescence measurements were carried out in an

Edinburgh spectrofluorimeter (FLS920) coupled with an Optistat DN cryostat (Oxford Instruments). The ITC temperature controller and a pressure gauge were used to realize the variable-temperature measurement in the range of 293-93 K. Spectra were collected at different temperatures after a 2-minute homeothermy.

Photoluminescence lifetime

Time-resolved photoluminescence lifetime was measured on an Edinburgh spectrofluorimeter (FLS920) with a time-correlated single-photon counting technique.

Photocurrent measurement

The photocurrent test was carried out on a CHI660E electrochemistry workstation. The 5 mg samples ([^{t}BuSAg]$_n$ or **SD/Ag90a**) and naphthol (0.5 wt. %, 10 μL) were dispersed in 0.5 mL ethanol, and the mixture was sonicated for about 30 min. Then a 100 μL solution was transferred by pipet dropped on the cleaned ITO glass, and the coated film was obtained after evaporation under ambient atmosphere. The prepared ITO glass film was used as working electrode, a Pt wire as the counter electrode, and an Ag/AgCl electrode as the reference electrode. A Na_2SO_4 aqueous solution (0.2 M) was used as the medium.

Supplementary Note.

Single crystals of **SD/Ag90a** and **SD/Ag90b** with appropriate dimensions were chosen under an optical microscope and quickly coated with high vacuum grease (Dow Corning Corporation) to prevent decomposition. Single-crystal X-ray diffraction data of **SD/Ag90a** and **SD/Ag90b** were collected on a Rigaku Oxford Diffraction XtaLAB Synergy diffractometer equipped with a HyPix-6000HE area detector at 100 K using Cu Kα (λ = 1.54184 Å) from a PhotonJet micro-focus X-ray Source. The diffraction images were processed and scaled using the *CrysAlis*^{Pro} software suite.[2] Both structures were solved using the charge-flipping algorithm, as implemented in the program *SUPERFLIP*[3] and refined by the full-matrix least-squares techniques against F_o^2 using the SHELXL program[4] through the OLEX2 interface.[5] Hydrogen atoms at carbon were placed in calculated positions and refined isotropically by using a riding model. Appropriate restraints and constraints were applied to the geometry and the atomic displacement parameters of the atoms in the cluster. All structures were examined using the Addsym subroutine of PLATON[6] to ensure that no additional symmetry could be applied to the models. Pertinent crystallographic data collection and refinement parameters are collated in Supplementary Table 1. Selected bond lengths and angles are collated in Supplementary Tables 2 and 3.

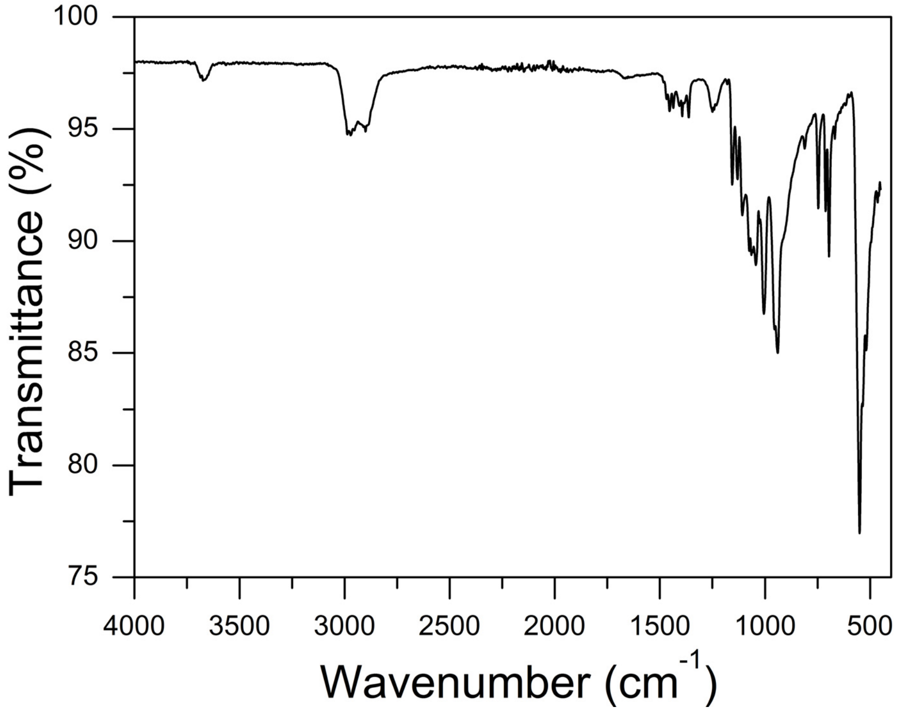

Supplementary Figure 1: IR spectrum of **SD/Ag90a**. Based on this spectrum, some peaks of characteristic groups were identified and assigned. The C-H stretching vibration peaks locate at *ca.* 2980 cm^{-1}. The symmetric and anti-symmetric stretching vibration peaks for PO_4^{3-} locate at 1007 and 1040 cm^{-1}, respectively. The aromatic C–C stretching vibration for $PhPO_3^{2-}$ is observed at 1150 cm^{-1}. The C–H out-of-plane bending vibration peaks for $PhPO_3^{2-}$ appeared at 689, 715, and 747 cm^{-1} as sharp peaks.

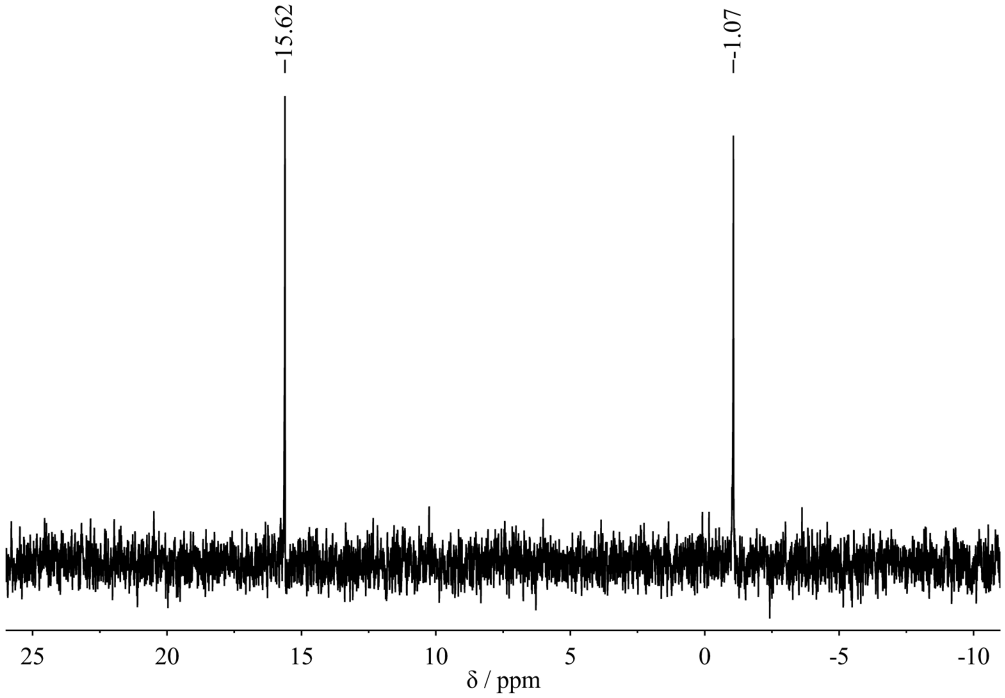

Supplementary Figure 2: ^{31}P NMR of **SD/Ag90a**. Crystals of **SD/Ag90a** were dissolved in DMSO and then digested with excess HCl (37%). The ^{31}P NMR of the digestion solution shows two sharp peaks with chemical shifts at δ = -1.07 and 15.62 ppm, corresponding to H_3PO_4 and $PhPO_3H_2$, respectively, which clearly verify the existence of two different P-containing chemicals in **SD/Ag90a**.

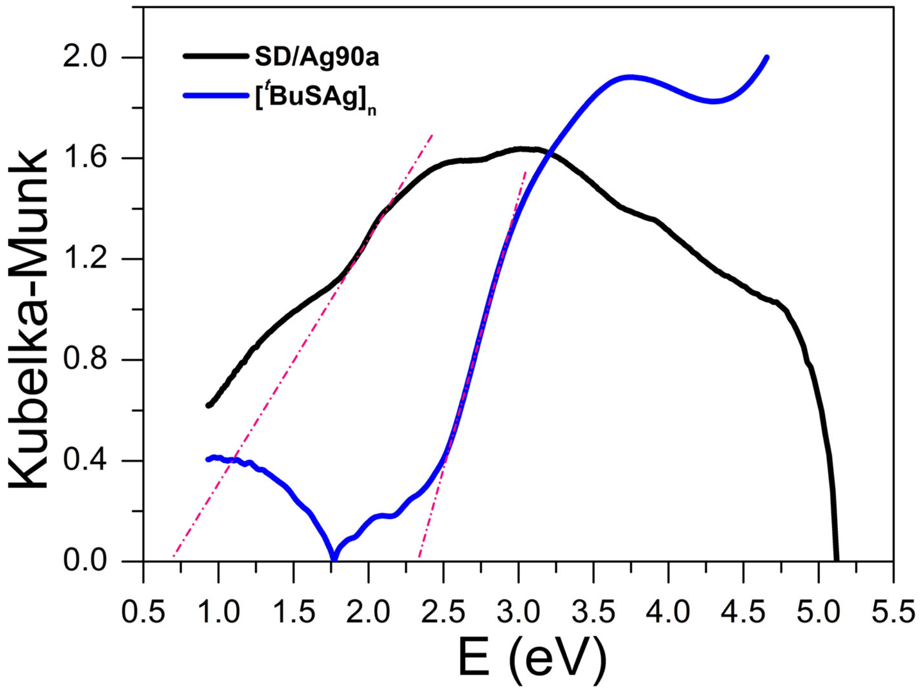

Supplementary Figure 3: Absorption spectra of **SD/Ag90a** and [^{t}BuSAg]$_n$ precursor derived from the diffuse reflectance spectra through the Kubelka–Munk function. The red dashed lines represent the extrapolation of the liner part of Kubelka–Munk function plots.

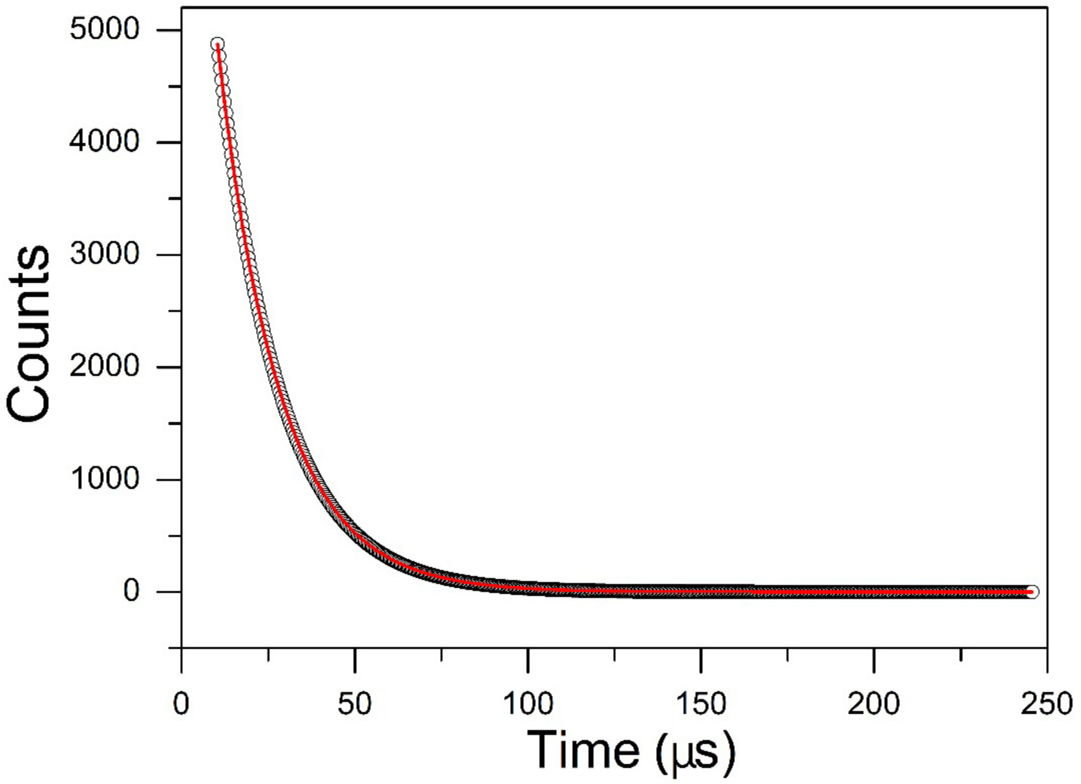

Supplementary Figure 4: The luminescence decay curve of **SD/Ag90a**. The emission lifetime of **SD/Ag90a**, falling in the microsecond scale $\tau_{\mathbf{SD/Ag90a}}$ = 17.80 μs at 93 K, suggests a triplet phosphorescence origin.

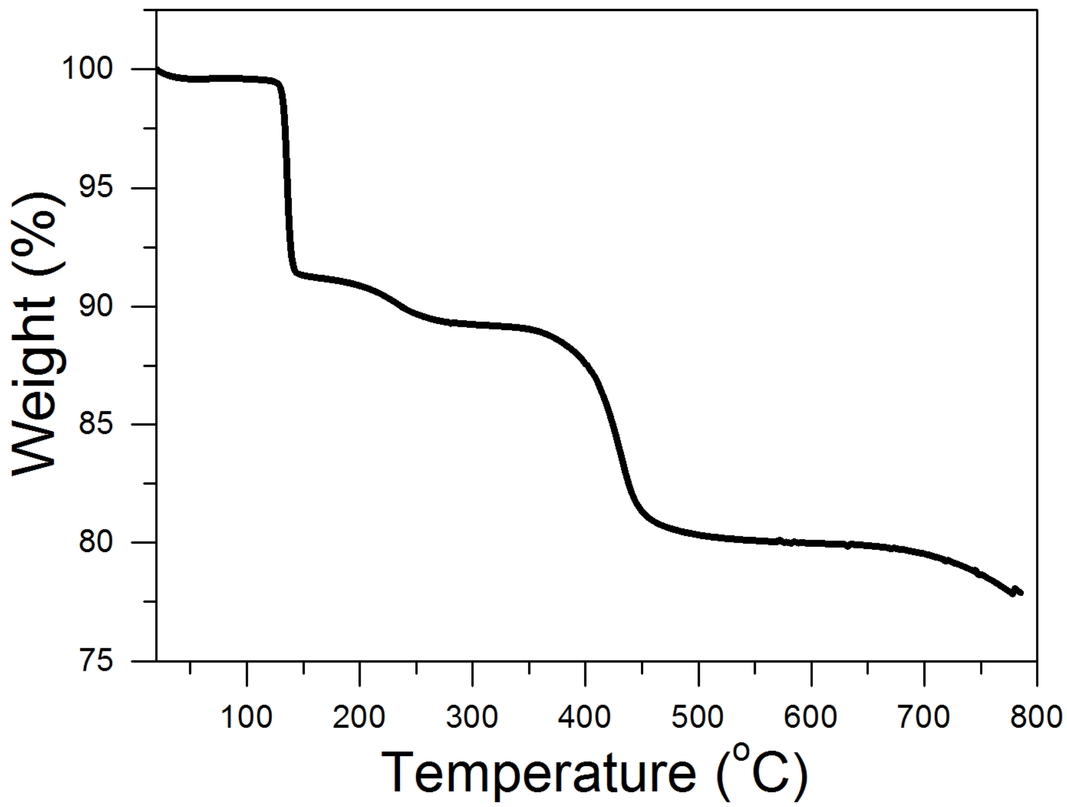

Supplementary Figure 5: The TGA (thermogravimetric analysis) for **SD/Ag90a.** This cluster can be stabilized to 125 ^{o}C; then, the ligands decompose upon further heating.

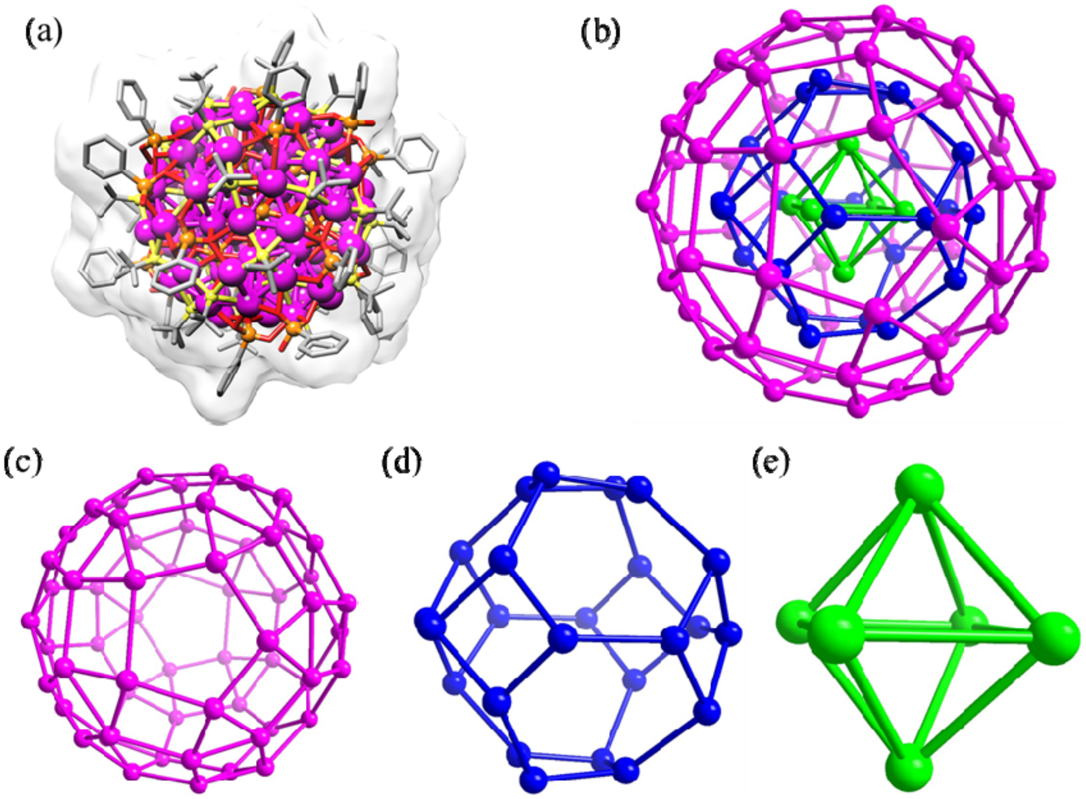

Supplementary Figure 6: The structural diagrams of **SD/Ag90b**. (**a**) Total cluster structure of **SD/Ag90b**. Hydrogen atoms are removed for clarity. Color legend: Ag, purple; P, brown; S, yellow; O, red; C, gray. (**b**) The ball-and-stick mode of the triply nested polyhedral silver skeleton viewed down the top silver pentagon. The different shells are individually colored. (**c**) The Ag_{60} rhombicosidodecahedron. (**d**) The Ag_{24} truncated octahedron. (**e**) The Ag_6 octahedron.

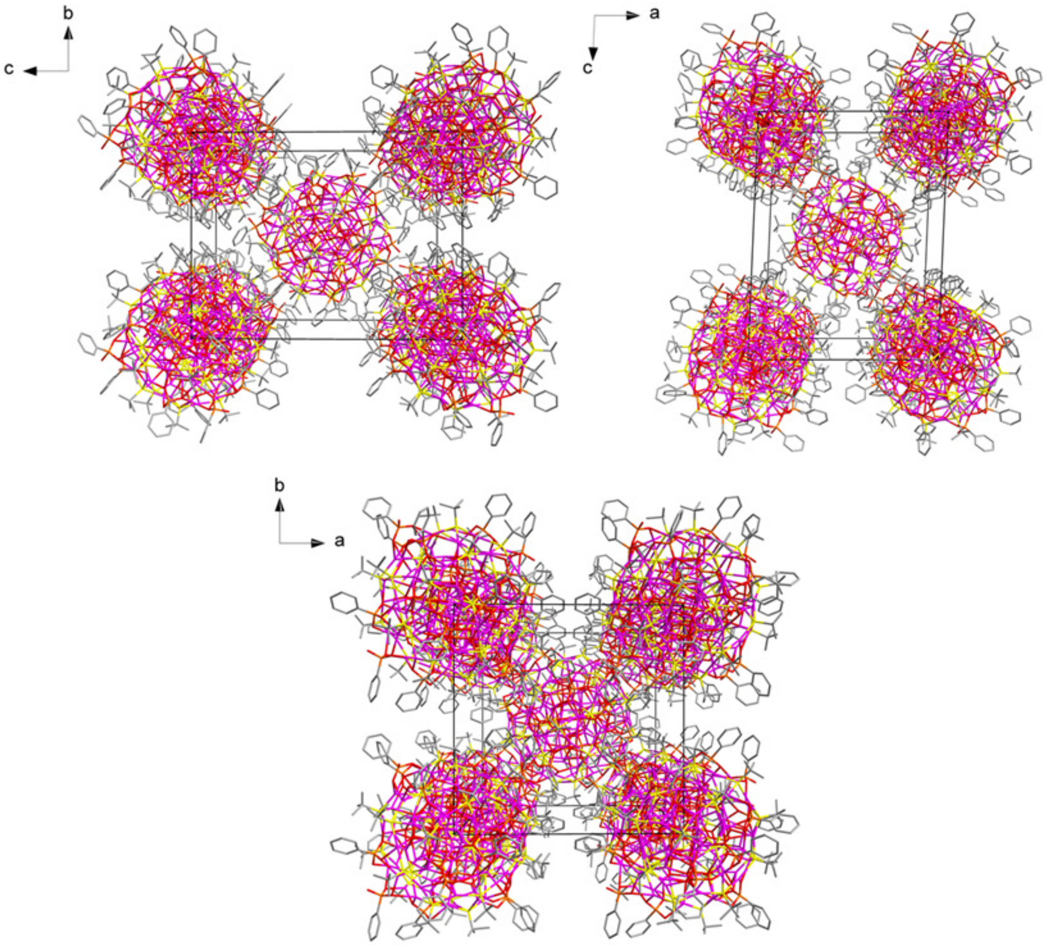

Supplementary Figure 7: Molecules packing in one unit cell for **SD/Ag90a** viewed along three different orientations.

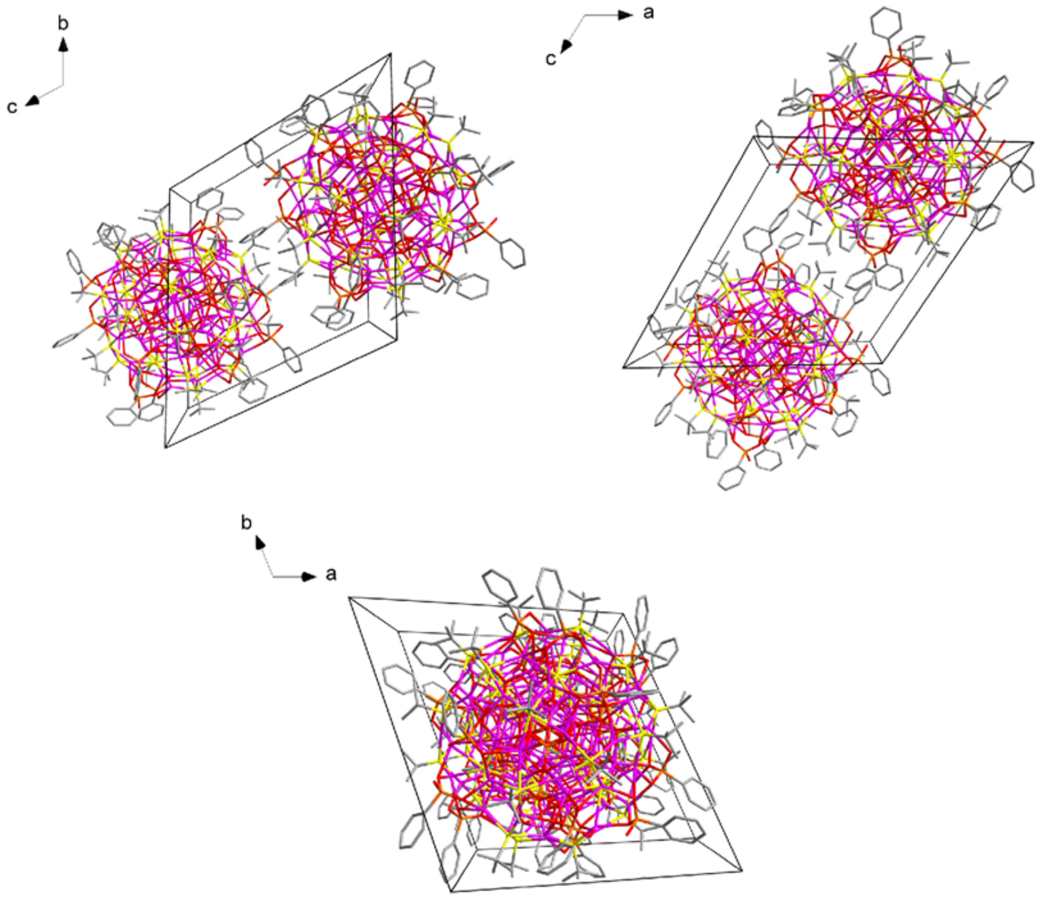

Supplementary Figure 8: Molecules packing in one unit cell for **SD/Ag90b** viewed along three different orientations.

(a)

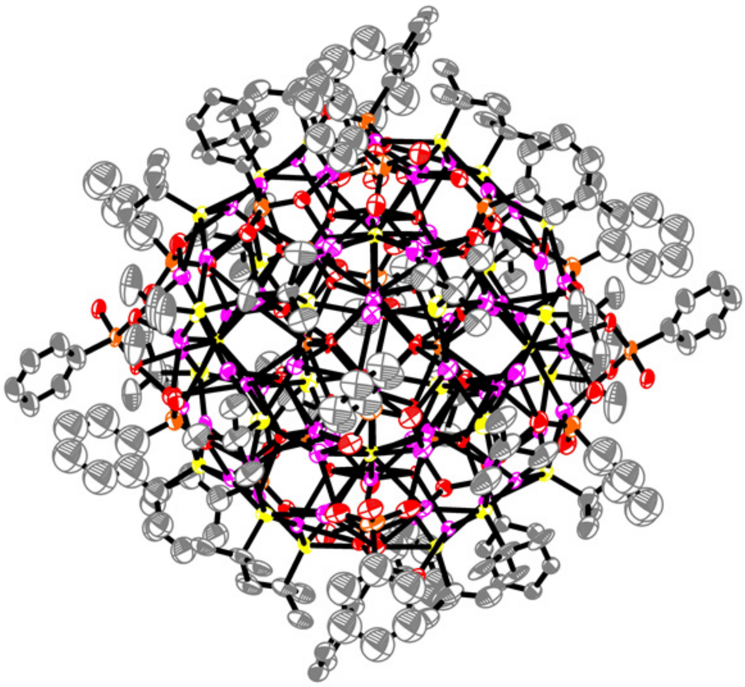

(b)

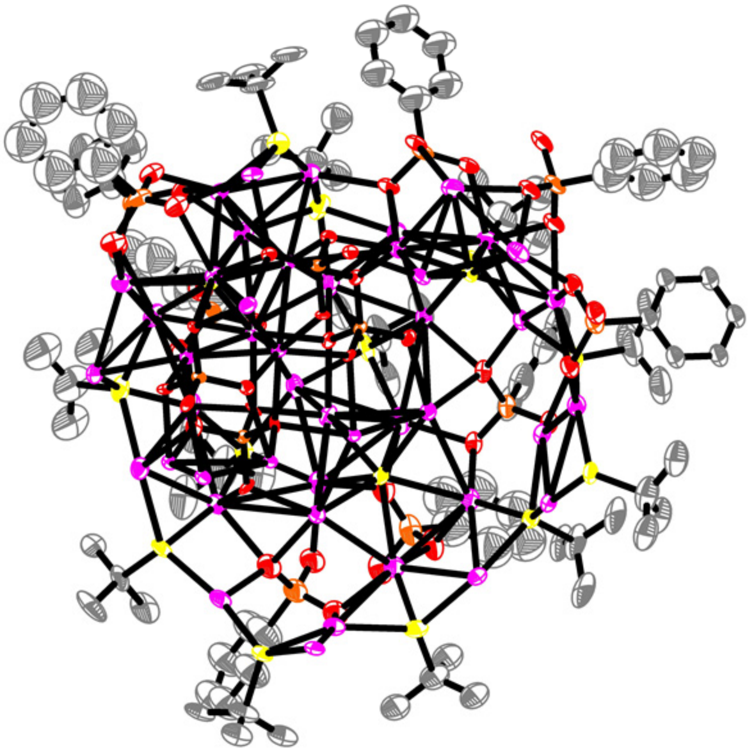

Supplementary Figure 9: ORTEP drawings (50% probability ellipsoids) of the total structure **(a)** and asymmetric unit structure **(b)** of **SD/Ag90a**. (Hydrogen atoms are omitted for clarity). Color legend: Ag, purple; P, brown; S, yellow; O, red; C, gray.

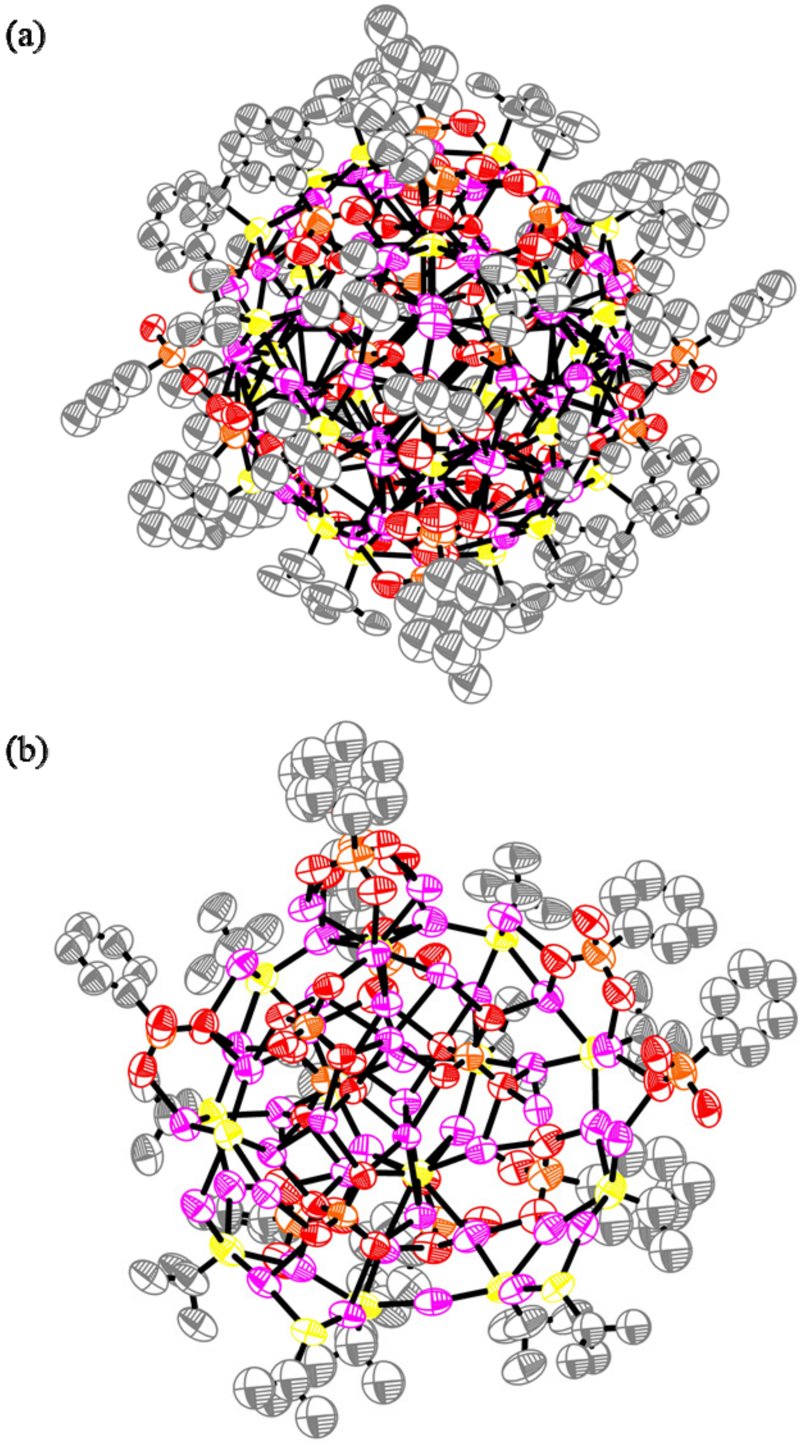

Supplementary Figure 10: ORTEP drawings (50% probability ellipsoids) of the total structure **(a)** and asymmetric unit structure **(b)** of **SD/Ag90b**. (Hydrogen atoms are omitted for clarity). Color legend: Ag, purple; P, brown; S, yellow; O, red; C, gray.

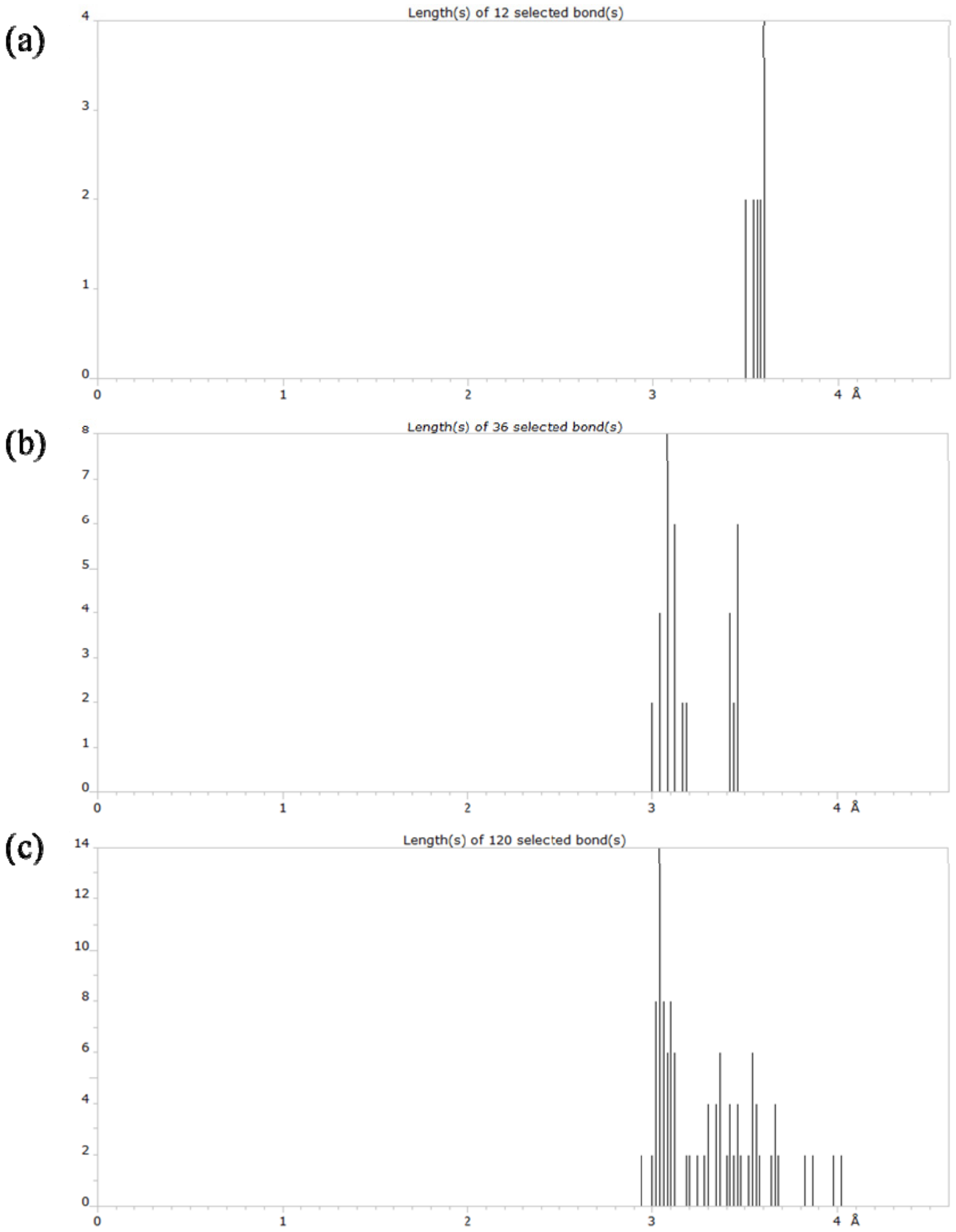

Supplementary Figure 11: Histogram of Ag-Ag edge lengths in three shells of **SD/Ag90a**. (**a**) The Ag-Ag edge lengths in the Ag_6 shell. (**b**) The Ag-Ag edge lengths in the Ag_{24} shell. (**c**) The Ag-Ag edge lengths in the Ag_{60} shell.

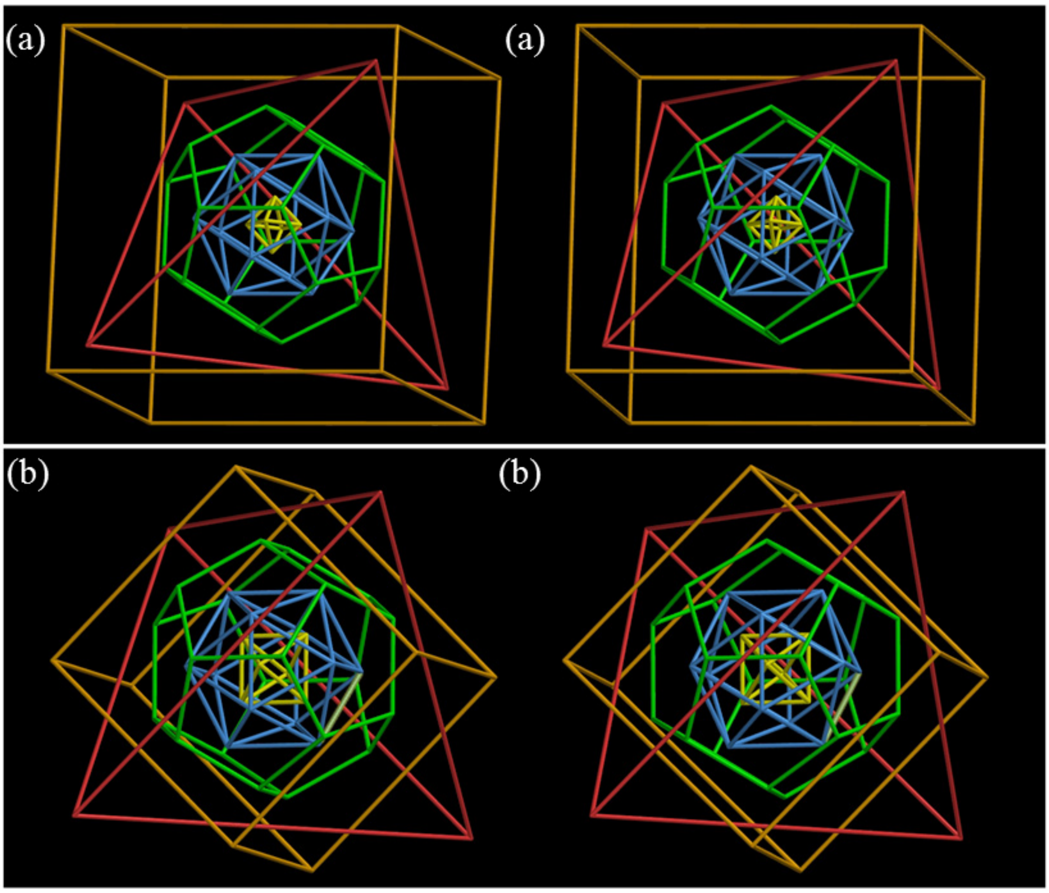

Supplementary Figure 12: Stereo view (**a** and **a**) of Kepler's Kosmos, corresponding to Fig. 4, and stereo view of the alternative alignment (**b** and **b**), corresponding to Fig. 5, in convergent (cross-eyed) stereo. The letters (**a**, **b** are shown both left and right in each of the two parts of the figure as an aid to convergence. Note that the orientation of the octahedron and the cube in (**b**) are shifted by 45º from their positions in (**a**).

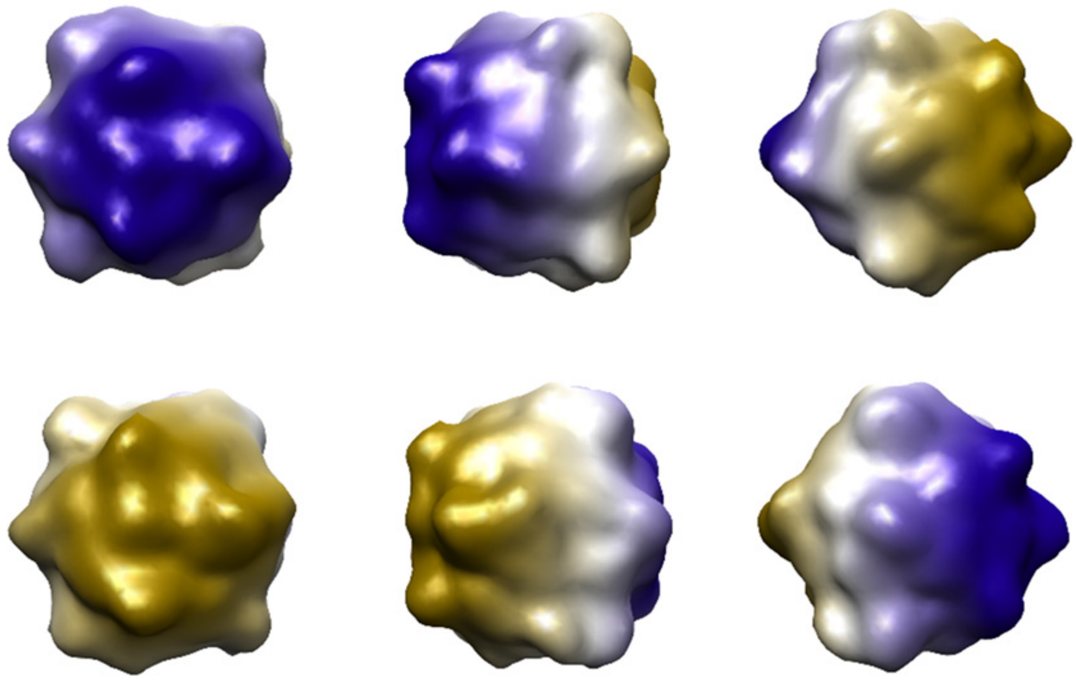

Supplementary Figure 13: The surface of **SD/Ag90a** calculated with the 3V Volume Assessor program[7] by rolling a virtual probe (1 Å) on the surface viewed along six different orientations

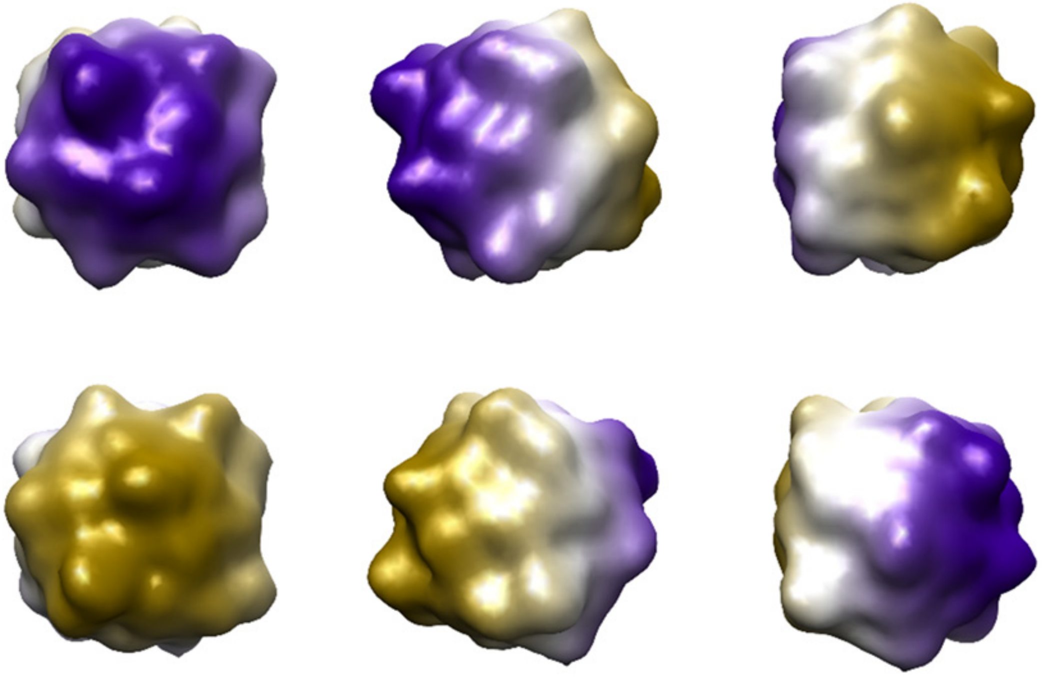

Supplementary Figure 14: The surface of **SD/Ag90b** calculated with the 3V Volume Assessor program[7] by rolling a virtual probe (1 Å) on the surface viewed along six different orientations.

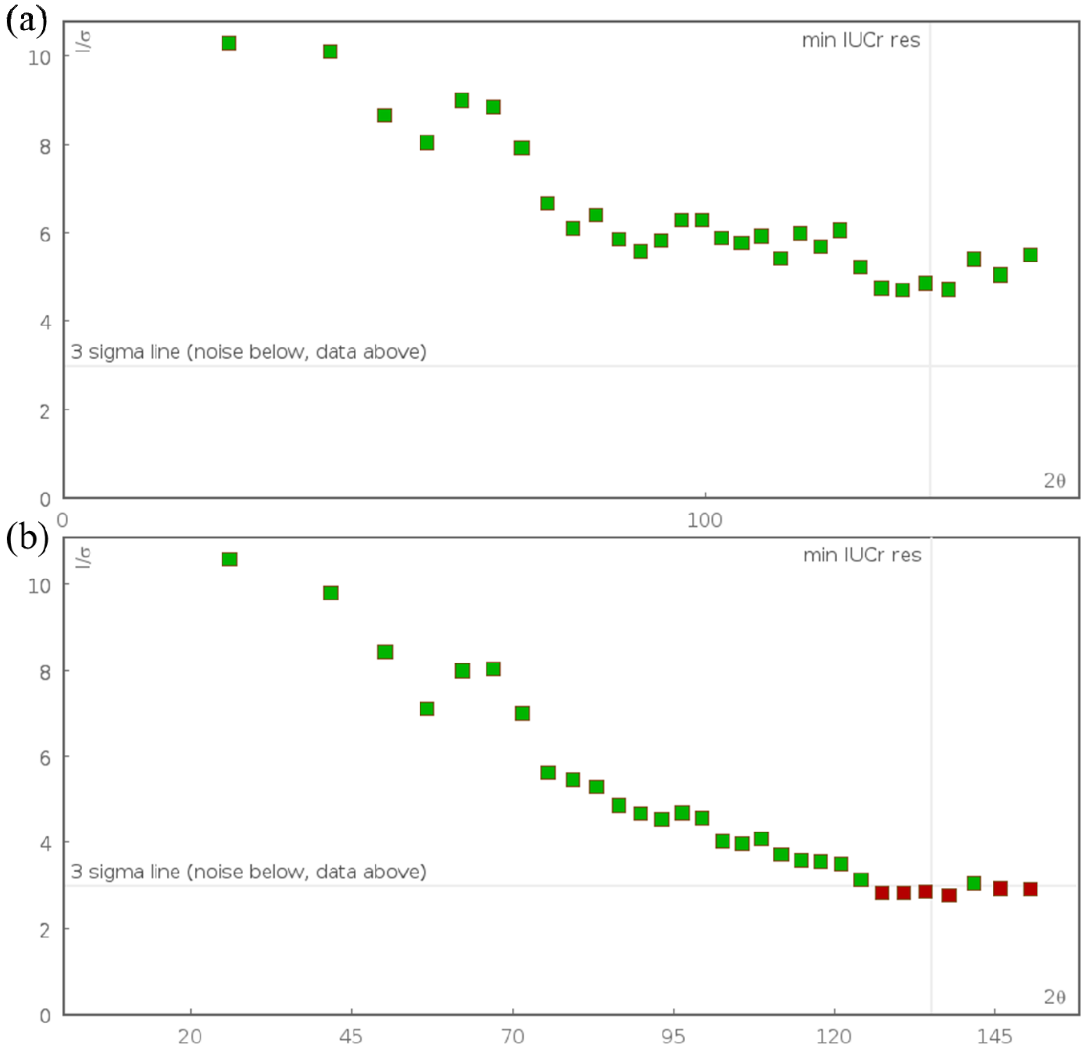

Supplementary Figure 15: The I/sigma vs. resolution plots of **SD/Ag90a** (**a**) and **SD/Ag90b** (**b**) derived from reflection data statistics using OLEX 1.2.10[5].

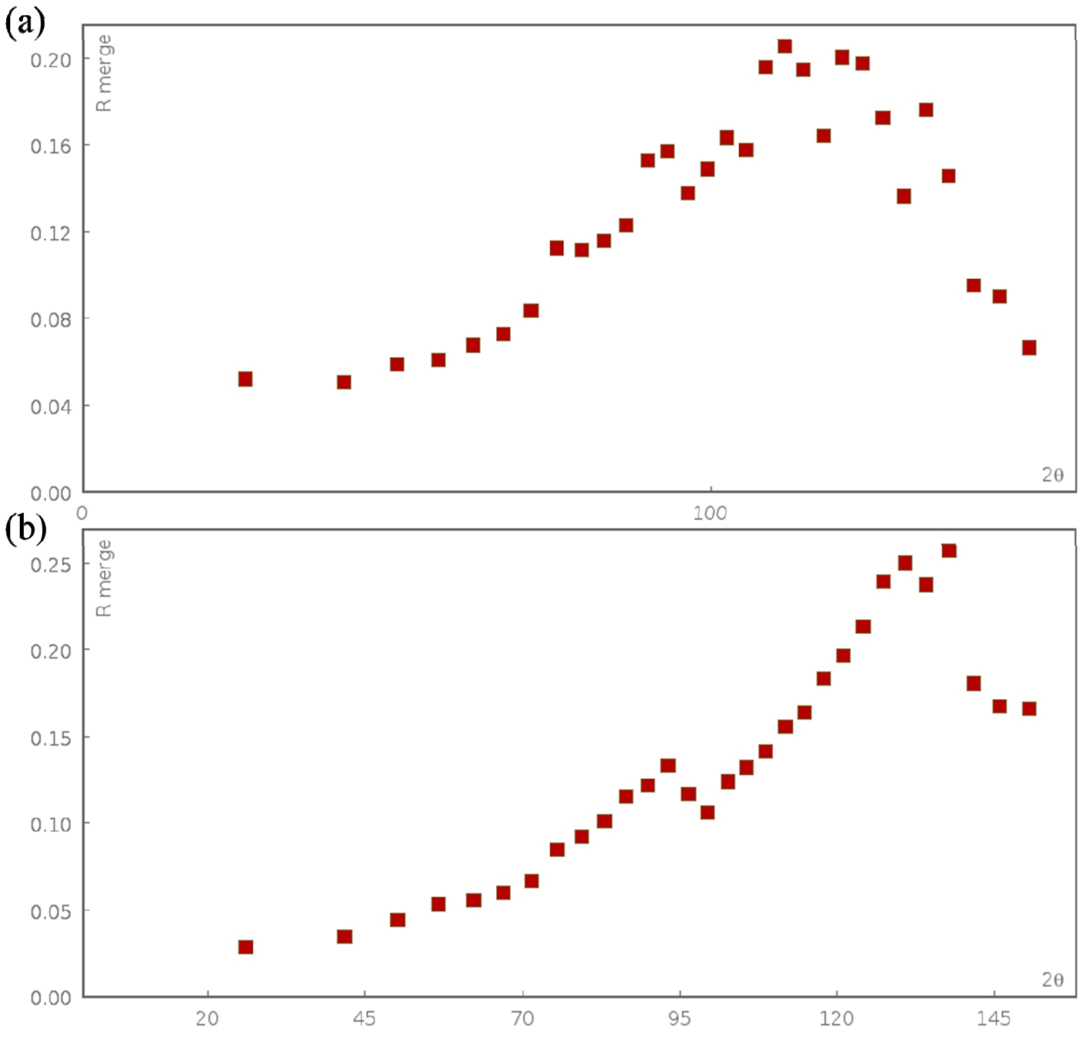

Supplementary Figure 16: The *R*merge vs. resolution plots of **SD/Ag90a** (**a**) and **SD/Ag90b** (**b**) derived from reflection data statistics using OLEX 1.2.10[5].

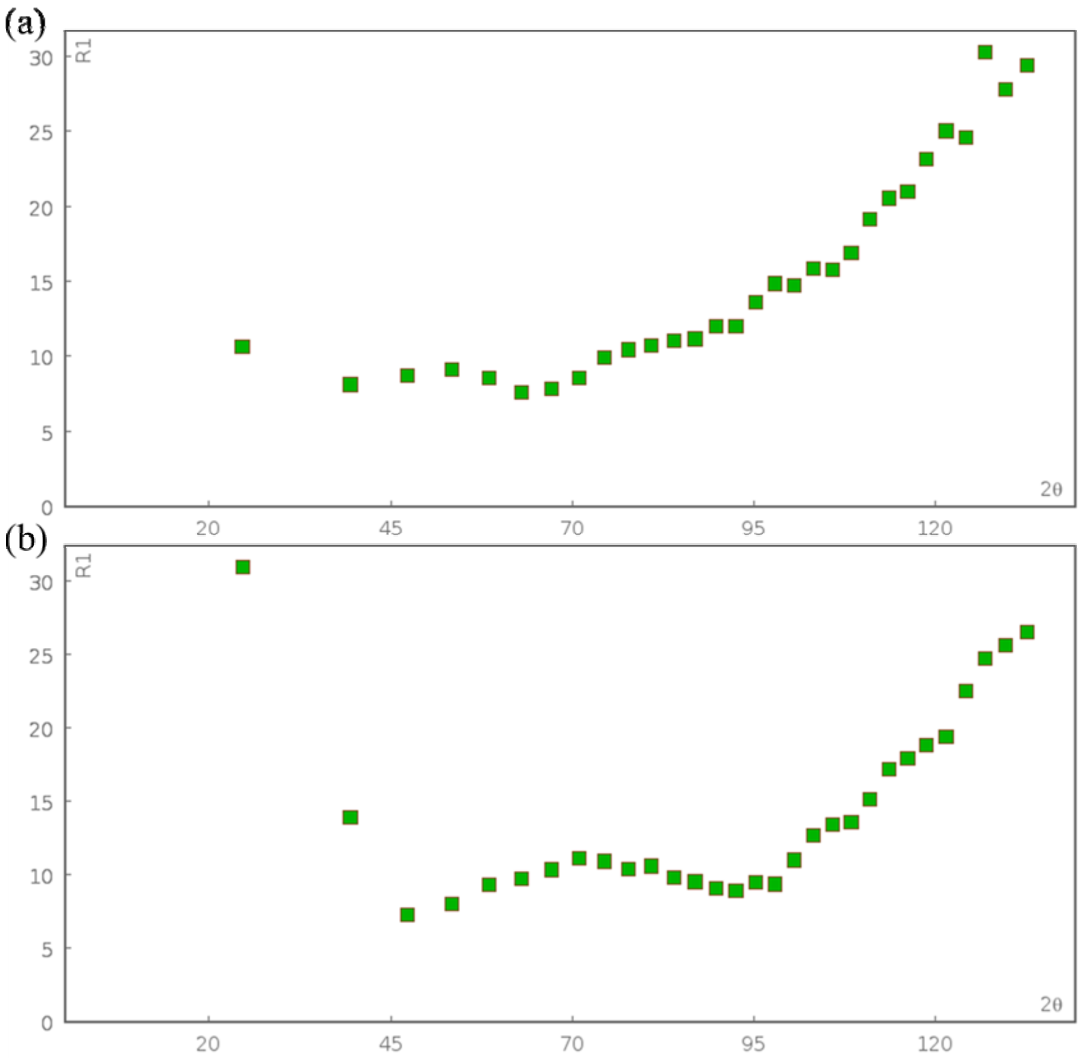

Supplementary Figure 17: The R_1 factor vs. resolution plots of **SD/Ag90a** (**a**) and **SD/Ag90b** (**b**) derived from reflection data statistics using OLEX 1.2.10[5].

Supplementary Table 1: Crystal data and structure refinements for **SD/Ag90a** and **SD/Ag90b**

	**SD/Ag90a**	**SD/Ag90b**
Empirical formula	$C_{204}H_{312}Ag_{90}O_{86}P_{26}S_{30}$	$C_{204}H_{312}Ag_{90}O_{86}P_{26}S_{30}$
Formula weight	15615.84	15615.84
Temperature/K	100.00(10)	99.99(10)
Crystal system	monoclinic	triclinic
Space group	$P2_1/n$	P-1
a/Å	24.1242(3)	22.3588(4)
g/Å	24.0789(3)	22.6664(4)
c/Å	31.5300(5)	23.3001(5)
α/°	90	110.5316(18)
β/°	92.4300(10)	115.488(2)
γ/°	90	99.7102(16)
Volume/$Å^3$	18298.8(4)	9241.2(4)
Z	2	1
$\rho_{calc}g/cm^3$	2.834	2.806
μ/mm^{-1}	40.797	40.392
F(000)	14648.0	7324.0
Radiation	CuKα (λ = 1.54184)	CuKα (λ = 1.54184)
Reflections collected	99002	94294
Independent reflections	32251 [R_{int}= 0.0995, R_{sigma}= 0.0791]	32640 [R_{int}= 0.0498, R_{sigma}= 0.0636]
Data/restraints/parameters	32251/820/1896	32640/495/1608
Goodness-of-fit on F^2	1.017	1.174
Final R indexes [I>=2σ (I)]	R_1= 0.0963, wR_2= 0.2357	R_1= 0.1288, wR_2= 0.2849
Final R indexes [all data]	R_1= 0.1305, wR_2= 0.2774	R_1= 0.1441, wR_2= 0.3102
Largest diff. peak/hole / e $Å^{-3}$	3.46/-3.44	3.67/-2.41

Supplementary Table 2: Selected bond distances (Å) and bond angles (°) for **SD/Ag90a** and **SD/Ag90b**

**SD/Ag90a**			
Ag1—Ag2	3.056 (3)	Ag22—Ag26	3.119 (3)
Ag1—Ag3	2.984 (2)	Ag22—Ag27	3.230 (3)
Ag1—Ag4	3.178 (2)	Ag22—Ag28	3.364 (3)
Ag1—Ag24^{i}	3.287 (3)	Ag22—S8	2.816 (6)
Ag1—Ag40^{i}	3.043 (3)	Ag22—S10	2.467 (8)
Ag1—S3	2.845 (5)	Ag22—O20	2.35 (2)
Ag1—S13^{i}	2.521 (7)	Ag22—O24	2.329 (15)
Ag1—O6	2.32 (4)	Ag23—Ag16^{i}	2.986 (2)
Ag1—O27	2.325 (19)	Ag23—Ag25	3.216 (3)
Ag2—Ag3	3.040 (2)	Ag23—Ag41	3.046 (2)
Ag2—Ag36^{i}	3.171 (2)	Ag23—S8	2.781 (5)
Ag2—Ag38^{i}	3.372 (3)	Ag23—S9	2.517 (8)
Ag2—Ag40^{i}	3.121 (3)	Ag23—O18	2.323 (19)
Ag2—S3	2.842 (5)	Ag23—O21	2.28 (2)
Ag2—S14^{i}	2.477 (7)	Ag24—Ag1^{i}	3.287 (3)
Ag2—O9	2.255 (18)	Ag24—Ag25	3.053 (3)
Ag2—O27	2.376 (19)	Ag24—Ag31^{i}	3.039 (3)
Ag3—Ag4	3.083 (2)	Ag24—S1^{i}	2.552 (7)
Ag3—Ag29^{i}	2.8639 (19)	Ag24—S13	2.525 (7)
Ag3—Ag36^{i}	3.139 (2)	Ag24—O6^{i}	2.45 (3)
Ag3—Ag40^{i}	2.957 (2)	Ag24—O29^{i}	2.494 (13)
Ag3—S3	2.453 (6)	Ag25—Ag31^{i}	3.137 (3)
Ag3—O28	2.326 (14)	Ag25—S9	2.512 (7)
Ag3—O36^{i}	2.321 (15)	Ag25—S13	2.508 (7)
Ag4—Ag6	3.134 (2)	Ag25—O18	2.50 (2)
Ag4—Ag29^{i}	3.139 (2)	Ag25—O28^{i}	2.530 (14)
Ag4—Ag45^{i}	3.184 (2)	Ag26—Ag27	3.007 (2)
Ag4—S3	2.466 (5)	Ag26—Ag28	3.043 (3)
Ag4—O7	2.52 (3)	Ag26—Ag41	3.131 (3)
Ag4—O29	2.353 (14)	Ag26—S8	2.749 (5)
Ag4—O42	2.367 (14)	Ag26—S12	2.481 (6)
Ag5—Ag6	3.076 (2)	Ag26—O16	2.361 (19)
Ag5—Ag7	3.190 (2)	Ag26—O20	2.33 (2)
Ag5—Ag8	3.301 (2)	Ag27—Ag28	2.933 (2)
Ag5—Ag36^{i}	3.139 (2)	Ag27—Ag41	3.120 (2)
Ag5—Ag45^{i}	3.113 (2)	Ag27—S8	2.432 (5)
Ag5—S3	2.889 (6)	Ag27—O39	2.328 (13)
Ag5—S5	2.463 (6)	Ag27—O40	2.293 (13)
Ag5—O9	2.317 (17)	Ag28—S10	2.467 (6)

Ag5—O25	2.325 (18)	Ag28—S12	2.487 (6)
Ag6—Ag7	3.027 (3)	Ag28—O8	2.33 (4)
Ag6—Ag14	3.363 (2)	Ag29—Ag3^{i}	2.8640 (19)
Ag6—Ag45^{i}	2.957 (2)	Ag29—Ag4^{i}	3.139 (2)
Ag6—S3	2.722 (6)	Ag29—Ag36	3.1661 (19)
Ag6—S4	2.516 (6)	Ag29—Ag45	2.866 (2)
Ag6—O5	2.33 (3)	Ag29—O30^{i}	2.447 (13)
Ag6—O25	2.363 (18)	Ag29—O35	2.438 (13)
Ag7—Ag45^{i}	2.938 (2)	Ag29—O38	2.386 (13)
Ag7—S4	2.482 (6)	Ag29—O43^{i}	2.405 (12)
Ag7—S5	2.450 (6)	Ag30—Ag35	2.8683 (19)
Ag7—O22	2.408 (16)	Ag30—Ag42	3.151 (2)
Ag8—Ag9	3.114 (2)	Ag30—Ag44	3.1789 (19)
Ag8—Ag10	3.093 (2)	Ag30—O30	2.424 (13)
Ag8—S5	2.492 (6)	Ag30—O35	2.389 (12)
Ag8—S6	2.526 (6)	Ag30—O38	2.397 (13)
Ag8—O2	2.44 (5)	Ag30—O43	2.394 (12)
Ag8—O34^{i}	2.558 (14)	Ag31—Ag24^{i}	3.039 (3)
Ag9—Ag10	3.056 (2)	Ag31—Ag25^{i}	3.137 (3)
Ag9—S6	2.510 (6)	Ag31—S1	2.475 (7)
Ag9—S7	2.499 (6)	Ag31—S9^{i}	2.524 (9)
Ag9—O13^{i}	2.43 (2)	Ag31—O15	2.47 (2)
Ag9—O32^{i}	2.488 (12)	Ag31—O31	2.474 (13)
Ag10—S5	2.589 (6)	Ag32—Ag33	2.957 (2)
Ag10—S7	2.578 (6)	Ag32—Ag34	3.026 (3)
Ag10—O23	2.351 (17)	Ag32—Ag35	2.974 (2)
Ag10—O33^{i}	2.514 (13)	Ag32—Ag44	3.279 (2)
Ag11—Ag12	3.108 (3)	Ag32—S6^{i}	2.569 (6)
Ag11—Ag13	3.165 (2)	Ag32—S11	2.777 (5)
Ag11—Ag16^{i}	2.961 (2)	Ag32—O12	2.360 (18)
Ag11—Ag23	3.069 (3)	Ag32—O13	2.23 (2)
Ag11—S7	2.497 (6)	Ag33—Ag34	3.240 (3)
Ag11—S8	2.827 (5)	Ag33—Ag35	2.936 (2)
Ag11—O21	2.380 (18)	Ag33—S6^{i}	2.441 (6)
Ag11—O24	2.299 (17)	Ag33—S15	2.464 (7)
Ag12—Ag16^{i}	2.942 (2)	Ag33—O2^{i}	2.41 (4)
Ag12—Ag23	3.111 (3)	Ag34—Ag35	3.116 (2)
Ag12—S7	2.431 (6)	Ag34—Ag37	3.301 (3)
Ag12—S9	2.448 (7)	Ag34—Ag42	3.075 (2)
Ag12—O15^{i}	2.41 (2)	Ag34—S11	2.814 (5)
Ag13—Ag16^{i}	3.092 (2)	Ag34—S15	2.469 (7)
Ag13—Ag20	3.185 (2)	Ag34—O3	2.41 (3)
Ag13—Ag21	3.380 (2)	Ag34—O12	2.388 (17)

Ag13—Ag22	3.050 (2)	Ag35—Ag42	3.056 (2)
Ag13—Ag27	3.083 (2)	Ag35—Ag44	3.137 (2)
Ag13—S8	2.530 (5)	Ag35—S11	2.450 (5)
Ag13—O22	2.500 (16)	Ag35—O34	2.310 (15)
Ag13—O33^{i}	2.353 (13)	Ag35—O37	2.282 (15)
Ag13—O41	2.351 (13)	Ag36—Ag2^{i}	3.171 (2)
Ag14—Ag19	3.032 (3)	Ag36—Ag3^{i}	3.139 (2)
Ag14—Ag21	3.086 (3)	Ag36—Ag5^{i}	3.139 (2)
Ag14—S2	2.522 (7)	Ag36—Ag45	3.017 (2)
Ag14—S4	2.503 (6)	Ag36—S3^{i}	2.516 (5)
Ag14—O7	2.34 (4)	Ag36—O2^{i}	2.45 (4)
Ag14—O42	2.462 (13)	Ag36—O34	2.364 (13)
Ag15—Ag17	3.017 (3)	Ag36—O37	2.371 (15)
Ag15—Ag18	2.937 (2)	Ag37—Ag38	3.098 (3)
Ag15—Ag43	3.138 (3)	Ag37—Ag39	3.075 (3)
Ag15—S1	2.498 (8)	Ag37—S12	2.547 (7)
Ag15—S2	2.456 (11)	Ag37—S15	2.489 (7)
Ag15—O7	2.40 (4)	Ag37—O4	2.24 (4)
Ag16—Ag11^{i}	2.961 (2)	Ag37—O39	2.487 (14)
Ag16—Ag12^{i}	2.942 (2)	Ag38—Ag2^{i}	3.372 (3)
Ag16—Ag13^{i}	3.092 (2)	Ag38—Ag39	3.059 (2)
Ag16—Ag20^{i}	2.866 (2)	Ag38—S14	2.521 (6)
Ag16—Ag23^{i}	2.986 (2)	Ag38—S15	2.587 (8)
Ag16—Ag41^{i}	3.172 (2)	Ag38—O1^{i}	2.54 (3)
Ag16—S8^{i}	2.434 (5)	Ag38—O37	2.490 (13)
Ag16—O31	2.328 (14)	Ag39—S12	2.470 (7)
Ag16—O32	2.301 (13)	Ag39—S14	2.526 (6)
Ag17—Ag18	2.987 (2)	Ag39—O17	2.47 (2)
Ag17—Ag31	3.354 (3)	Ag39—O36	2.465 (14)
Ag17—Ag43	3.072 (3)	Ag40—Ag1^{i}	3.043 (3)
Ag17—Ag44	3.156 (2)	Ag40—Ag2^{i}	3.121 (3)
Ag17—S1	2.520 (7)	Ag40—Ag3^{i}	2.957 (2)
Ag17—S11	2.801 (5)	Ag40—S13	2.440 (7)
Ag17—O11	2.344 (18)	Ag40—S14	2.461 (7)
Ag17—O14	2.34 (2)	Ag40—O17	2.47 (2)
Ag18—Ag30	2.8678 (19)	Ag41—Ag16^{i}	3.172 (2)
Ag18—Ag42	3.089 (2)	Ag41—S8	2.469 (5)
Ag18—Ag43	3.045 (2)	Ag41—O17	2.450 (18)
Ag18—Ag44	3.046 (2)	Ag41—O28^{i}	2.366 (14)
Ag18—S11	2.443 (5)	Ag41—O36	2.365 (13)
Ag18—O29	2.319 (14)	Ag42—Ag43	3.086 (2)
Ag18—O42	2.343 (13)	Ag42—S11	2.511 (5)
Ag19—Ag21	3.054 (2)	Ag42—O39	2.339 (12)

Ag19—S2	2.585 (10)	Ag42—O40	2.351 (13)
Ag19—S10	2.633 (8)	Ag43—S2	2.481 (7)
Ag19—O8	2.58 (4)	Ag43—S11	2.785 (5)
Ag19—O40	2.441 (13)	Ag43—O3	2.20 (3)
Ag20—Ag16^{i}	2.866 (2)	Ag43—O11	2.394 (17)
Ag20—Ag27	2.858 (2)	Ag44—S11	2.477 (5)
Ag20—Ag41	3.2400 (19)	Ag44—O31	2.344 (14)
Ag20—O30^{i}	2.410 (13)	Ag44—O32	2.377 (13)
Ag20—O35^{i}	2.421 (12)	Ag45—Ag4^{i}	3.184 (2)
Ag20—O38	2.433 (12)	Ag45—Ag5^{i}	3.113 (2)
Ag20—O43	2.414 (12)	Ag45—Ag6^{i}	2.957 (2)
Ag21—Ag22	3.346 (3)	Ag45—Ag7^{i}	2.939 (2)
Ag21—S4	2.522 (6)	Ag45—S3^{i}	2.449 (5)
Ag21—S10	2.468 (6)	Ag45—O33	2.312 (13)
Ag21—O22	2.449 (16)	Ag45—O41^{i}	2.361 (13)
Ag21—O41	2.476 (14)		
S13^{i}—Ag1—S3	136.52 (19)	S9—Ag23—S8	138.0 (2)
O6—Ag1—S3	112.1 (10)	O18—Ag23—S8	119.4 (5)
O6—Ag1—S13^{i}	84.5 (10)	O18—Ag23—S9	83.9 (6)
O6—Ag1—O27	117.3 (10)	O21—Ag23—S8	80.1 (6)
O27—Ag1—S3	77.7 5)	O21—Ag23—S9	125.4 (6)
O27—Ag1—S13^{i}	131.5 (6)	O21—Ag23—O18	112.7 (7)
S14^{i}—Ag2—S3	132.41 (18)	S13—Ag24—S1^{i}	156.1 (2)
O9—Ag2—S3	81.6 (4)	O6^{i}—Ag24—S1^{i}	112.8 (9)
O9—Ag2—S14^{i}	128.0 (5)	O6^{i}—Ag24—S13	81.7 (9)
O9—Ag2—O27	86.6 (7)	O6^{i}—Ag24—O29^{i}	104.6 (9)
O27—Ag2—S3	77.0 (5)	O29^{i}—Ag24—S1^{i}	81.3 (3)
O27—Ag2—S14^{i}	132.4 (5)	O29^{i}—Ag24—S13	114.4 (3)
O28—Ag3—S3	136.1 (4)	S9—Ag25—O28^{i}	110.7 (3)
O36^{i}—Ag3—S3	135.3 (3)	S13—Ag25—S9	155.0 (3)
O36^{i}—Ag3—O28	86.4 (5)	S13—Ag25—O28^{i}	82.5 (3)
S3—Ag4—O7	124.1 (8)	O18—Ag25—S9	80.5 (5)
O29—Ag4—S3	131.9 (4)	O18—Ag25—S13	116.2 (5)
O29—Ag4—O7	93.1 (9)	O18—Ag25—O28^{i}	109.0 (6)
O29—Ag4—O42	85.4 (5)	S12—Ag26—S8	132.71 (18)
O42—Ag4—S3	130.5 (3)	O16—Ag26—S8	104.5 (5)
O42—Ag4—O7	73.5 (9)	O16—Ag26—S12	106.9 (5)
S5—Ag5—S3	130.15 (17)	O20—Ag26—S8	80.3 (5)
O9—Ag5—S3	79.6 (4)	O20—Ag26—S12	135.8 (6)
O9—Ag5—S5	133.5 (5)	O20—Ag26—O16	86.2 (8)
O9—Ag5—O25	86.4 (7)	O39—Ag27—S8	135.3 (3)
O25—Ag5—S3	76.4 (5)	O40—Ag27—S8	138.5 (3)
O25—Ag5—S5	130.3 (6)	O40—Ag27—O39	84.1 (4)

S4—Ag6—S3	137.93 (17)	S10—Ag28—S12	156.6 (2)
O5—Ag6—S3	103.1 (10)	O8—Ag28—S10	94.8 (10)
O5—Ag6—S4	106.3 (10)	O8—Ag28—S12	108.1 (10)
O5—Ag6—O25	87.3 (10)	O35—Ag29—O30^{i}	134.1 (4)
O25—Ag6—S3	79.2 (5)	O38—Ag29—O30^{i}	81.3 (4)
O25—Ag6—S4	131.2 (6)	O38—Ag29—O35	79.2 (4)
S5—Ag7—S4	160.5 (2)	O38—Ag29—O43^{i}	130.4 (4)
O22—Ag7—S4	89.2 (4)	O43^{i}—Ag29—O30^{i}	81.4 (4)
O22—Ag7—S5	110.1 (4)	O43^{i}—Ag29—O35	80.4 (4)
S5—Ag8—S6	157.4 (2)	O35—Ag30—O30	82.9 (4)
S5—Ag8—O34^{i}	110.3 (3)	O35—Ag30—O38	80.0 (4)
S6—Ag8—O34^{i}	81.0 (3)	O35—Ag30—O43	133.5 (4)
O2—Ag8—S5	117.0 (10)	O38—Ag30—O30	134.5 (4)
O2—Ag8—S6	84.6 (10)	O43—Ag30—O30	82.1 (4)
O2—Ag8—O34^{i}	73.2 (10)	O43—Ag30—O38	80.0 (4)
S7—Ag9—S6	158.2 (2)	S1—Ag31—S9^{i}	155.2 (2)
O13^{i}—Ag9—S6	78.1 (5)	O15—Ag31—S1	115.6 (6)
O13^{i}—Ag9—S7	116.5 (5)	O15—Ag31—S9^{i}	85.6 (6)
O13^{i}—Ag9—O32^{i}	104.0 (6)	O15—Ag31—O31	79.6 (5)
O32^{i}—Ag9—S6	114.9 (4)	O31—Ag31—S1	113.3 (4)
O32^{i}—Ag9—S7	78.7 (4)	O31—Ag31—S9^{i}	81.8 (4)
S7—Ag10—S5	159.57 (19)	S6^{i}—Ag32—S11	135.23 (17)
O23—Ag10—S5	96.5 (4)	O12—Ag32—S6^{i}	135.5 (4)
O23—Ag10—S7	95.3 (4)	O12—Ag32—S11	79.6 (4)
O23—Ag10—O33^{i}	119.4 (5)	O13—Ag32—S6^{i}	80.5 (6)
O33^{i}—Ag10—S5	82.5 (3)	O13—Ag32—S11	108.9 (6)
O33^{i}—Ag10—S7	106.0 (3)	O13—Ag32—O12	118.5 (7)
S7—Ag11—S8	132.04 (18)	S6^{i}—Ag33—S15	161.6 (2)
O21—Ag11—S7	130.7 (6)	O2^{i}—Ag33—S6^{i}	87.3 (11)
O21—Ag11—S8	77.6 (5)	O2^{i}—Ag33—S15	110.9 (11)
O24—Ag11—S7	124.1 (4)	S15—Ag34—S11	130.78 (19)
O24—Ag11—S8	86.7 (4)	O3—Ag34—S11	83.3 (7)
O24—Ag11—O21	90.1 (7)	O3—Ag34—S15	131.9 (8)
S7—Ag12—S9	161.4 (3)	O12—Ag34—S11	78.4(4)
O15^{i}—Ag12—S7	110.0 (5)	O12—Ag34—S15	127.5 (5)
O15^{i}—Ag12—S9	88.6 (6)	O12—Ag34—O3	86.7 (9)
O22—Ag13—S8	125.8 (4)	O34—Ag35—S11	135.8 (4)
O33^{i}—Ag13—S8	129.6 (4)	O37—Ag35—S11	137.2 (4)
O33^{i}—Ag13—O22	90.6 (5)	O37—Ag35—O34	85.5 (5)
O41—Ag13—S8	130.2 (3)	O2^{i}—Ag36—S3^{i}	126.1 (10)
O41—Ag13—O22	80.3 (5)	O34—Ag36—S3^{i}	132.6 (4)
O41—Ag13—O33^{i}	84.4 (4)	O34—Ag36—O2^{i}	76.5 (11)
S4—Ag14—S2	157.2 (3)	O34—Ag36—O37	82.3 (5)

O7—Ag14—S2	83.3 (9)	O37—Ag36—S3^{i}	131.2 (4)
O7—Ag14—S4	116.3 (9)	O37—Ag36—O2^{i}	89.9 (11)
O7—Ag14—O42	75.0 (8)	S15—Ag37—S12	156.8 (2)
O42—Ag14—S2	80.9 (3)	O4—Ag37—S12	97.5 (10)
O42—Ag14—S4	114.1 (3)	O4—Ag37—S15	91.6 (10)
S2—Ag15—S1	163.2 (3)	O4—Ag37—O39	122.8 (9)
O7—Ag15—S1	112.8 (10)	O39—Ag37—S12	81.9 (3)
O7—Ag15—S2	83.6 (10)	O39—Ag37—S15	110.8 (3)
O31—Ag16—S8^{i}	133.8 (4)	S14—Ag38—S15	155.9 (2)
O32—Ag16—S8^{i}	138.1 (3)	S14—Ag38—O1^{i}	88.2 (8)
O32—Ag16—O31	86.7 (5)	O1^{i}—Ag38—S15	102.0 (8)
S1—Ag17—S11	135.3 (2)	O37—Ag38—S14	112.4 (4)
O11—Ag17—S1	133.2 (5)	O37—Ag38—S15	81.0 (4)
O11—Ag17—S11	78.5 (4)	O37—Ag38—O1^{i}	121.4 (8)
O14—Ag17—S1	107.9 (6)	S12—Ag39—S14	157.5 (2)
O14—Ag17—S11	103.8 (5)	O17—Ag39—S12	112.7 (5)
O14—Ag17—O11	86.7 (7)	O17—Ag39—S14	86.6 (5)
O29—Ag18—S11	136.7 (4)	O36—Ag39—S12	111.9 (4)
O29—Ag18—O42	86.7 (5)	O36—Ag39—S14	82.5 (4)
O42—Ag18—S11	135.1 (4)	O36—Ag39—O17	78.3 (5)
S2—Ag19—S10	158.0 (2)	S13—Ag40—S14	162.5 (2)
O8—Ag19—S2	114.3 (9)	S13—Ag40—O17	109.2 (5)
O8—Ag19—S10	85.3 (9)	S14—Ag40—O17	88.0 (5)
O40—Ag19—S2	109.1 (4)	O17—Ag41—S8	124.2 (5)
O40—Ag19—S10	82.7 (3)	O28^{i}—Ag41—S8	129.2 (4)
O40—Ag19—O8	81.1 (9)	O28^{i}—Ag41—O17	93.0 (6)
O30^{i}—Ag20—O35^{i}	82.5 (4)	O36—Ag41—S8	130.3 (4)
O30^{i}—Ag20—O38	81.1 (4)	O36—Ag41—O17	80.6 (6)
O30^{i}—Ag20—O43	133.7 (4)	O36—Ag41—O28^{i}	84.5 (5)
O35^{i}—Ag20—O38	131.8 (4)	O39—Ag42—S11	133.0 (3)
O43—Ag20—O35^{i}	80.6 (4)	O39—Ag42—O40	82.6 (5)
O43—Ag20—O38	78.9 (4)	O40—Ag42—S11	133.4 (3)
S10—Ag21—S4	156.1 (2)	S2—Ag43—S11	133.2 (2)
S10—Ag21—O41	111.3 (3)	O3—Ag43—S2	124.9 (8)
O22—Ag21—S4	87.4 (4)	O3—Ag43—S11	87.9 (8)
O22—Ag21—S10	112.7 (4)	O3—Ag43—O11	88.3 (10)
O22—Ag21—O41	78.9 (5)	O11—Ag43—S2	128.6 (5)
O41—Ag21—S4	84.4 (3)	O11—Ag43—S11	78.1 (4)
S10—Ag22—S8	125.56 (19)	O31—Ag44—S11	133.5 (4)
O20—Ag22—S8	78.5 (5)	O31—Ag44—O32	84.6 (5)
O20—Ag22—S10	126.0 (6)	O32—Ag44—S11	129.4 (4)
O24—Ag22—S8	86.4 (4)	O33—Ag45—S3^{i}	140.4 (3)
O24—Ag22—S10	132.6 (4)	O33—Ag45—O41^{i}	85.1 (5)

O24—Ag22—O20	90.8 (7)	O41^{i}—Ag45—S3^{i}	132.2 (3)
Symmetry code: (i) −*x*+1, −*y*+1, −*z*+1.			
**SD/Ag90b**			
Ag1—Ag2	3.033 (3)	Ag20—S15	2.777 (5)
Ag1—Ag3	3.109 (2)	Ag20—O20	2.296 (18)
Ag1—Ag16	3.361 (3)	Ag20—O24	2.34 (2)
Ag1—Ag27	3.099 (2)	Ag21—Ag23	3.193 (2)
Ag1—Ag39	3.248 (3)	Ag21—Ag24	3.0473 (19)
Ag1—S2	2.458 (7)	Ag21—Ag25	3.1981 (18)
Ag1—S3	2.810 (5)	Ag21—S15	2.479 (5)
Ag1—O2^{i}	2.38 (2)	Ag21—O23	2.555 (19)
Ag1—O6^{i}	2.291 (18)	Ag21—O29	2.354 (12)
Ag2—Ag4	3.189 (2)	Ag21—O38^{i}	2.379 (12)
Ag2—Ag27	3.074 (2)	Ag22—Ag3^{i}	3.085 (2)
Ag2—Ag39	3.072 (3)	Ag22—Ag4^{i}	3.208 (2)
Ag2—S3	2.860 (5)	Ag22—Ag26^{i}	2.8618 (18)
Ag2—S4	2.481 (6)	Ag22—Ag36	2.981 (2)
Ag2—O2^{i}	2.352 (17)	Ag22—S3^{i}	2.434 (5)
Ag2—O15	2.345 (16)	Ag22—O29	2.285 (12)
Ag3—Ag10^{i}	3.033 (2)	Ag22—O38^{i}	2.325 (12)
Ag3—Ag22^{i}	3.085 (2)	Ag23—Ag24	3.0818 (19)
Ag3—Ag26	3.1761 (18)	Ag23—Ag37	3.121 (3)
Ag3—Ag27	3.1156 (19)	Ag23—Ag44	3.054 (2)
Ag3—S3	2.517 (5)	Ag23—S9	2.469 (5)
Ag3—O5^{i}	2.529 (18)	Ag23—S15	2.818 (5)
Ag3—O28^{i}	2.350 (12)	Ag23—O19	2.341 (17)
Ag3—O34	2.333 (12)	Ag23—O22	2.369 (18)
Ag4—Ag11^{i}	3.163 (2)	Ag24—Ag25	2.8607 (17)
Ag4—Ag22^{i}	3.208 (2)	Ag24—Ag37	3.255 (2)
Ag4—Ag26	3.1676 (18)	Ag24—Ag38	3.0925 (19)
Ag4—Ag27	3.036 (2)	Ag24—Ag44	2.9070 (19)
Ag4—S3	2.463 (5)	Ag24—S15	2.422 (4)
Ag4—O13	2.448 (14)	Ag24—O31	2.314 (11)
Ag4—O39	2.370 (12)	Ag24—O40	2.339 (13)
Ag4—O41	2.338 (13)	Ag25—Ag38	3.1784 (18)
Ag5—Ag6	3.097 (3)	Ag25—O30	2.405 (11)
Ag5—Ag7	3.210 (3)	Ag25—O33	2.417 (11)
Ag5—Ag40	3.344 (3)	Ag25—O37^{i}	2.385 (11)
Ag5—Ag42	2.951 (2)	Ag25—O42	2.360 (10)
Ag5—S6	2.479 (6)	Ag26—Ag22^{i}	2.8619 (18)
Ag5—S11^{i}	2.446 (6)	Ag26—Ag27	2.8466 (17)
Ag5—O13	2.442 (19)	Ag26—O30^{i}	2.403 (11)
Ag6—Ag7	3.080 (2)	Ag26—O33	2.401 (10)

Ag6—Ag13^{i}	3.291 (3)	Ag26—O37	2.390 (10)
Ag6—Ag31	3.230 (2)	Ag26—O42	2.438 (11)
Ag6—Ag42	2.9419 (19)	Ag27—Ag39	2.9012 (19)
Ag6—S7	2.742 (5)	Ag27—S3	2.438 (5)
Ag6—S11^{i}	2.551 (7)	Ag27—O35	2.315 (12)
Ag6—O9	2.40 (2)	Ag27—O43	2.308 (13)
Ag6—O12	2.37 (2)	Ag28—Ag37	3.356 (3)
Ag7—Ag32	3.093 (2)	Ag28—Ag40	3.063 (2)
Ag7—Ag42	3.179 (2)	Ag28—Ag43	2.997 (2)
Ag7—S6	2.482 (7)	Ag28—S4	2.575 (6)
Ag7—S7	2.824 (5)	Ag28—S5	2.462 (6)
Ag7—O12	2.32 (2)	Ag28—O16	2.47 (2)
Ag7—O26	2.31 (2)	Ag28—O43	2.514 (12)
Ag8—Ag9	2.980 (3)	Ag29—Ag30	2.8451 (17)
Ag8—Ag30	2.9758 (19)	Ag29—Ag31	3.1429 (16)
Ag8—Ag31	3.184 (2)	Ag29—Ag32	3.1806 (17)
Ag8—Ag33	3.022 (2)	Ag29—Ag42	2.8624 (17)
Ag8—S1^{i}	2.495 (7)	Ag29—O30	2.419 (11)
Ag8—S7	2.723 (5)	Ag29—O33^{i}	2.379 (10)
Ag8—O7	2.268 (18)	Ag29—O37	2.423 (11)
Ag8—O10	2.46 (2)	Ag29—O42	2.388 (10)
Ag9—Ag30	2.912 (2)	Ag30—Ag31	3.037 (2)
Ag9—Ag33	3.172 (3)	Ag30—Ag32	3.0605 (19)
Ag9—S1^{i}	2.464 (6)	Ag30—Ag33	3.124 (2)
Ag9—S8	2.475 (6)	Ag30—S7	2.466 (5)
Ag9—O5	2.32 (2)	Ag30—O28	2.311 (12)
Ag10—Ag3^{i}	3.033 (2)	Ag30—O34^{i}	2.350 (12)
Ag10—Ag11	3.153 (3)	Ag31—Ag42	3.036 (2)
Ag10—Ag22	3.073 (2)	Ag31—S7	2.474 (5)
Ag10—Ag35	3.305 (3)	Ag31—O8	2.501 (17)
Ag10—Ag36	3.236 (3)	Ag31—O32^{i}	2.351 (12)
Ag10—S3^{i}	2.834 (5)	Ag31—O36	2.348 (13)
Ag10—S14	2.469 (6)	Ag32—Ag33	3.090 (2)
Ag10—O3	2.314 (16)	Ag32—Ag42	3.0668 (19)
Ag10—O6	2.311 (19)	Ag32—S7	2.516 (4)
Ag11—Ag4^{i}	3.163 (2)	Ag32—O27	2.58 (2)
Ag11—Ag12	3.193 (3)	Ag32—O31	2.345 (11)
Ag11—Ag22	2.926 (2)	Ag32—O40	2.336 (12)
Ag11—Ag36	3.098 (3)	Ag33—Ag34	3.360 (2)
Ag11—S3^{i}	2.758 (5)	Ag33—S7	2.803 (5)
Ag11—S10	2.554 (8)	Ag33—S8	2.454 (7)
Ag11—O3	2.316 (19)	Ag33—O10	2.41 (2)
Ag11—O14^{i}	2.373 (18)	Ag33—O26	2.26 (3)

Ag12—Ag13	3.081 (3)	Ag34—Ag35	3.044 (2)
Ag12—Ag45	3.040 (2)	Ag34—Ag41	3.036 (2)
Ag12—S10	2.571 (7)	Ag34—S8	2.496 (6)
Ag12—S11	2.521 (7)	Ag34—S9	2.587 (5)
Ag12—O14^{i}	2.390 (19)	Ag34—O25	2.33 (2)
Ag12—O39^{i}	2.511 (11)	Ag34—O31	2.517 (11)
Ag13—Ag6^{i}	3.291 (3)	Ag35—Ag41	3.016 (2)
Ag13—Ag45	3.094 (3)	Ag35—S8	2.586 (7)
Ag13—S11	2.530 (7)	Ag35—S14	2.506 (7)
Ag13—S13	2.492 (7)	Ag35—O5	2.50 (2)
Ag13—O9^{i}	2.51 (2)	Ag35—O28	2.479 (11)
Ag13—O36^{i}	2.506 (11)	Ag36—S10	2.444 (7)
Ag14—Ag18	3.151 (3)	Ag36—S14	2.487 (7)
Ag14—Ag19	2.9438 (18)	Ag36—O23	2.40 (2)
Ag14—Ag20	3.064 (3)	Ag37—Ag38	3.092 (2)
Ag14—S12	2.444 (8)	Ag37—S5	2.448 (7)
Ag14—S13	2.420 (8)	Ag37—S15	2.810 (5)
Ag14—O8^{i}	2.42 (2)	Ag37—O18	2.30 (2)
Ag15—Ag16	3.054 (3)	Ag37—O19	2.340 (17)
Ag15—Ag17	3.063 (3)	Ag38—S15	2.504 (5)
Ag15—S1	2.446 (6)	Ag38—O16	2.58 (2)
Ag15—S12	2.499 (6)	Ag38—O35	2.366 (12)
Ag15—O8^{i}	2.48 (2)	Ag38—O43	2.346 (12)
Ag15—O32	2.466 (12)	Ag39—S2	2.440 (7)
Ag16—Ag17	3.021 (2)	Ag39—S4	2.498 (7)
Ag16—S1	2.560 (7)	Ag39—O16	2.37 (2)
Ag16—S2	2.510 (7)	Ag40—Ag43	2.987 (2)
Ag16—O4^{i}	2.34 (2)	Ag40—S4	2.429 (7)
Ag16—O34	2.529 (12)	Ag40—S6	2.507 (7)
Ag17—S2	2.552 (8)	Ag40—O13	2.482 (17)
Ag17—S12	2.528 (8)	Ag40—O41	2.489 (13)
Ag17—O17	2.325 (19)	Ag41—S9	2.446 (6)
Ag17—O35	2.501 (11)	Ag41—S14	2.522 (6)
Ag18—Ag19	2.995 (2)	Ag41—O23	2.49 (2)
Ag18—Ag20	3.027 (3)	Ag41—O29	2.490 (11)
Ag18—Ag38	3.071 (2)	Ag42—S7	2.432 (5)
Ag18—S12	2.458 (7)	Ag42—O39	2.332 (12)
Ag18—S15	2.791 (5)	Ag42—O41	2.285 (13)
Ag18—O18	2.30 (2)	Ag43—S5	2.615 (7)
Ag18—O20	2.365 (17)	Ag43—S6	2.522 (6)
Ag19—Ag20	2.925 (2)	Ag43—O27	2.53 (3)
Ag19—Ag21	3.139 (2)	Ag43—O40	2.460 (12)
Ag19—Ag25	2.8365 (16)	Ag44—S5	2.510 (7)

Ag19—Ag38	3.090 (2)	Ag44—S9	2.483 (6)
Ag19—S15	2.434 (5)	Ag44—O27	2.46 (3)
Ag19—O32	2.312 (12)	Ag45—S10	2.482 (8)
Ag19—O36^{i}	2.337 (12)	Ag45—S13	2.526 (8)
Ag20—Ag21	3.159 (2)	Ag45—O24	2.36 (2)
Ag20—Ag45	3.287 (3)	Ag45—O38^{i}	2.533 (11)
Ag20—S13	2.552 (7)		
S2—Ag1—S3	129.95 (18)	S9—Ag23—S15	128.73 (14)
O2^{i}—Ag1—S2	131.3 (5)	O19—Ag23—S9	140.9 (5)
O2^{i}—Ag1—S3	78.2 (5)	O19—Ag23—S15	78.6 (4)
O6^{i}—Ag1—S2	127.4 (5)	O19—Ag23—O22	88.2 (7)
O6^{i}—Ag1—S3	86.8 (5)	O22—Ag23—S9	106.1 (5)
O6^{i}—Ag1—O2^{i}	86.3 (6)	O22—Ag23—S15	105.8 (5)
S4—Ag2—S3	128.64 (17)	O31—Ag24—S15	138.0 (3)
O2^{i}—Ag2—S3	77.6 (4)	O31—Ag24—O40	83.2 (4)
O2^{i}—Ag2—S4	142.4 (5)	O40—Ag24—S15	136.3 (3)
O15—Ag2—S3	104.4 (4)	O30—Ag25—O33	132.1 (3)
O15—Ag2—S4	107.3 (4)	O37^{i}—Ag25—O30	80.2 (4)
O15—Ag2—O2^{i}	86.9 (7)	O37^{i}—Ag25—O33	81.3 (4)
S3—Ag3—O5^{i}	128.0 (5)	O42—Ag25—O30	80.7 (4)
O28^{i}—Ag3—S3	131.2 (3)	O42—Ag25—O33	80.0 (4)
O28^{i}—Ag3—O5^{i}	77.1 (5)	O42—Ag25—O37^{i}	132.3 (3)
O34—Ag3—S3	129.9 (3)	O30^{i}—Ag26—O42	130.9 (4)
O34—Ag3—O5^{i}	89.2 (6)	O33—Ag26—O30^{i}	80.5 (3)
O34—Ag3—O28^{i}	83.8 (4)	O33—Ag26—O42	78.8 (4)
O13—Ag4—S3	123.0 (4)	O37—Ag26—O30^{i}	80.2 (4)
O39—Ag4—S3	126.9 (3)	O37—Ag26—O33	131.5 (4)
O39—Ag4—O13	96.0 (5)	O37—Ag26—O42	81.3 (4)
O41—Ag4—S3	134.4 (3)	O35—Ag27—S3	136.9 (3)
O41—Ag4—O13	79.8 (5)	O43—Ag27—S3	136.3 (3)
O41—Ag4—O39	82.3 (4)	O43—Ag27—O35	84.2 (4)
S11^{i}—Ag5—S6	160.0 (3)	S5—Ag28—S4	157.7 (2)
O13—Ag5—S6	94.4 (4)	S5—Ag28—O16	112.6 (5)
O13—Ag5—S11^{i}	105.4 (4)	S5—Ag28—O43	113.0 (3)
S11^{i}—Ag6—S7	132.91 (16)	O16—Ag28—S4	86.9 (5)
O9—Ag6—S7	113.0 (5)	O16—Ag28—O43	79.5 (5)
O9—Ag6—S11^{i}	86.1 (5)	O43—Ag28—S4	80.0 (3)
O12—Ag6—S7	79.2 (5)	O30—Ag29—O37	133.6 (4)
O12—Ag6—S11^{i}	132.1 (6)	O33^{i}—Ag29—O30	80.6 (4)
O12—Ag6—O9	116.2 (7)	O33^{i}—Ag29—O37	81.3 (4)
S6—Ag7—S7	131.93 (17)	O33^{i}—Ag29—O42	132.4 (4)
O12—Ag7—S6	128.7 (6)	O42—Ag29—O30	79.9 (4)
O12—Ag7—S7	78.4 (6)	O42—Ag29—O37	81.6 (4)

O26—Ag7—S6	123.0 (7)	O28—Ag30—S7	137.1 (3)
O26—Ag7—S7	83.0 (6)	O28—Ag30—O34^{i}	84.3 (4)
O26—Ag7—O12	97.2 (9)	O34^{i}—Ag30—S7	137.4 (3)
S1^{i}—Ag8—S7	134.48 (17)	S7—Ag31—O8	119.0 (5)
O7—Ag8—S1^{i}	107.1 (6)	O32^{i}—Ag31—S7	133.2 (3)
O7—Ag8—S7	102.9 (6)	O32^{i}—Ag31—O8	79.4 (5)
O7—Ag8—O10	84.8 (7)	O36—Ag31—S7	131.3 (3)
O10—Ag8—S1^{i}	136.4 (6)	O36—Ag31—O8	94.3 (6)
O10—Ag8—S7	79.0 (6)	O36—Ag31—O32^{i}	84.0 (4)
S1^{i}—Ag9—S8	160.5 (2)	S7—Ag32—O27	123.3 (6)
O5—Ag9—S1^{i}	107.1 (6)	O31—Ag32—S7	132.8 (3)
O5—Ag9—S8	92.2 (6)	O31—Ag32—O27	89.1 (6)
S14—Ag10—S3^{i}	133.68 (18)	O40—Ag32—S7	133.0 (3)
O3—Ag10—S3^{i}	76.3 (5)	O40—Ag32—O27	78.0 (7)
O3—Ag10—S14	125.7 (6)	O40—Ag32—O31	82.6 (4)
O6—Ag10—S3^{i}	85.8 (4)	S8—Ag33—S7	132.91 (18)
O6—Ag10—S14	129.0 (5)	O10—Ag33—S7	78.3 (5)
O6—Ag10—O3	89.0 (7)	O10—Ag33—S8	129.6 (6)
S10—Ag11—S3^{i}	136.58 (18)	O26—Ag33—S7	84.3 (5)
O3—Ag11—S3^{i}	77.8 (5)	O26—Ag33—S8	123.2 (6)
O3—Ag11—S10	128.6 (5)	O26—Ag33—O10	93.4 (9)
O3—Ag11—O14^{i}	117.4 (6)	S8—Ag34—S9	158.2 (2)
O14^{i}—Ag11—S3^{i}	114.9 (4)	S8—Ag34—O31	111.1 (3)
O14^{i}—Ag11—S10	85.1 (5)	O25—Ag34—S8	97.7 (7)
S11—Ag12—S10	157.3 (2)	O25—Ag34—S9	92.8 (7)
O14^{i}—Ag12—S10	84.4 (5)	O25—Ag34—O31	115.1 (6)
O14^{i}—Ag12—S11	110.0 (5)	O31—Ag34—S9	81.1 (3)
O14^{i}—Ag12—O39^{i}	109.2 (5)	S14—Ag35—S8	155.59 (19)
O39^{i}—Ag12—S10	113.2 (3)	S14—Ag35—O5	115.6 (5)
O39^{i}—Ag12—S11	79.4 (3)	O5—Ag35—S8	85.6 (5)
S13—Ag13—S11	157.4 (2)	O28—Ag35—S8	83.0 (3)
S13—Ag13—O9^{i}	111.4 (5)	O28—Ag35—S14	112.9 (3)
S13—Ag13—O36^{i}	79.7 (3)	O28—Ag35—O5	75.4 (5)
O9^{i}—Ag13—S11	84.2 (5)	S10—Ag36—S14	160.6 (2)
O36^{i}—Ag13—S11	111.5 (3)	O23—Ag36—S10	106.6 (5)
O36^{i}—Ag13—O9^{i}	109.0 (5)	O23—Ag36—S14	92.6 (5)
S13—Ag14—S12	160.9 (2)	S5—Ag37—S15	124.89 (17)
O8^{i}—Ag14—S12	90.6 (6)	O18—Ag37—S5	130.1 (6)
O8^{i}—Ag14—S13	108.4 (6)	O18—Ag37—S15	87.2 (5)
S1—Ag15—S12	157.5 (2)	O18—Ag37—O19	91.1 (7)
S1—Ag15—O8^{i}	111.2 (5)	O19—Ag37—S5	128.6 (5)
S1—Ag15—O32	113.1 (3)	O19—Ag37—S15	78.8 (4)
O8^{i}—Ag15—S12	88.0 (5)	S15—Ag38—O16	124.7 (4)

O32—Ag15—S12	81.6 (3)	O35—Ag38—S15	131.0 (3)
O32—Ag15—O8^{i}	77.8 (5)	O35—Ag38—O16	90.4 (5)
S2—Ag16—S1	159.8 (2)	O43—Ag38—S15	131.6 (3)
S2—Ag16—O34	109.1 (3)	O43—Ag38—O16	80.4 (6)
O4^{i}—Ag16—S1	95.2 (5)	O43—Ag38—O35	82.3 (4)
O4^{i}—Ag16—S2	95.1 (5)	S2—Ag39—S4	156.7 (2)
O4^{i}—Ag16—O34	119.2 (6)	O16—Ag39—S2	112.0 (6)
O34—Ag16—S1	80.7 (3)	O16—Ag39—S4	90.9 (6)
S12—Ag17—S2	159.7 (2)	S4—Ag40—S6	157.4 (2)
O17—Ag17—S2	96.2 (6)	S4—Ag40—O13	105.9 (5)
O17—Ag17—S12	93.8 (6)	S4—Ag40—O41	111.6 (3)
O17—Ag17—O35	120.1 (6)	O13—Ag40—S6	92.7 (5)
O35—Ag17—S2	81.9 (3)	O13—Ag40—O41	76.3 (4)
O35—Ag17—S12	108.0 (3)	O41—Ag40—S6	84.8 (3)
S12—Ag18—S15	134.70 (16)	S9—Ag41—S14	160.1 (2)
O18—Ag18—S12	123.9 (5)	S9—Ag41—O23	107.5 (5)
O18—Ag18—S15	87.6 (5)	S9—Ag41—O29	108.9 (3)
O18—Ag18—O20	90.1 (7)	O23—Ag41—S14	89.7 (5)
O20—Ag18—S12	126.4 (5)	O29—Ag41—S14	84.3 (3)
O20—Ag18—S15	79.3 (4)	O29—Ag41—O23	77.6 (5)
O32—Ag19—S15	137.0 (3)	O39—Ag42—S7	138.5 (3)
O32—Ag19—O36^{i}	85.1 (4)	O41—Ag42—S7	135.9 (3)
O36^{i}—Ag19—S15	136.5 (4)	O41—Ag42—O39	84.3 (4)
S13—Ag20—S15	135.76 (16)	S6—Ag43—S5	158.3 (2)
O20—Ag20—S13	128.7 (5)	O27—Ag43—S5	90.1 (6)
O20—Ag20—S15	80.7 (5)	O27—Ag43—S6	109.3 (6)
O20—Ag20—O24	118.0 (6)	O40—Ag43—S5	83.9 (3)
O24—Ag20—S13	82.6 (6)	O40—Ag43—S6	109.4 (3)
O24—Ag20—S15	114.8 (5)	O40—Ag43—O27	76.7 (6)
S15—Ag21—O23	123.7 (5)	S9—Ag44—S5	154.9 (2)
O29—Ag21—S15	132.2 (3)	O27—Ag44—S5	94.1 (7)
O29—Ag21—O23	78.9 (5)	O27—Ag44—S9	110.0 (7)
O29—Ag21—O38^{i}	84.0 (4)	S10—Ag45—S13	158.4 (2)
O38^{i}—Ag21—S15	128.5 (4)	S10—Ag45—O38^{i}	82.0 (3)
O38^{i}—Ag21—O23	94.3 (5)	S13—Ag45—O38^{i}	110.3 (3)
O29—Ag22—S3^{i}	137.4 (3)	O24—Ag45—10	111.4 (6)
O29—Ag22—O38^{i}	86.8 (4)	O24—Ag45—S13	82.8 (6)
O38^{i}—Ag22—S3^{i}	133.6 (3)	O24—Ag45—O38^{i}	106.8 (6)
Symmetry code: (i) −*x*+1, −*y*+1, −*z*.			

Supplementary Table 3: The Ag-Ag edge lengths in Ag_{60}, Ag_{24} and Ag_{6} shells of **SD/Ag90a**

**Ag-Ag edge lengths in Ag_{60} shell of SD/Ag90a (Å)**					
Ag1—Ag2	3.06	Ag22—Ag28	3.37	$Ag8^{i}$—$Ag9^{i}$	3.12
Ag1—Ag6	3.99	Ag23—Ag25	3.22	$Ag8^{i}$—$Ag10^{i}$	3.10
Ag1—$Ag24^{i}$	3.29	Ag23—Ag26	3.88	$Ag9^{i}$—$Ag10^{i}$	3.06
Ag1—$Ag40^{i}$	3.04	Ag24—$Ag1^{i}$	3.29	$Ag9^{i}$—$Ag12^{i}$	3.65
Ag2—$Ag38^{i}$	3.37	Ag24—$Ag31^{i}$	3.04	$Ag10^{i}$—$Ag11^{i}$	3.44
Ag2—$Ag40^{i}$	3.12	Ag24—$Ag15^{i}$	3.66	$Ag11^{i}$—$Ag12^{i}$	3.11
Ag2—Ag5	3.83	Ag24—Ag25	3.05	$Ag11^{i}$—$Ag23^{i}$	3.07
Ag5—Ag6	3.08	Ag25—Ag40	3.55	$Ag11^{i}$—$Ag22^{i}$	3.67
Ag5—Ag7	3.19	Ag25—$Ag31^{i}$	3.14	$Ag12^{i}$—$Ag23^{i}$	3.11
Ag5—Ag8	3.30	Ag26—Ag28	3.04	$Ag14^{i}$—$Ag19^{i}$	3.03
Ag6—Ag7	3.03	Ag26—Ag39	3.43	$Ag14^{i}$—$Ag15^{i}$	3.59
Ag6—Ag14	3.36	Ag28—Ag37	3.57	$Ag14^{i}$—$Ag21^{i}$	3.09
Ag7—Ag10	3.57	Ag31—$Ag25^{i}$	3.14	$Ag15^{i}$—$Ag43^{i}$	3.14
Ag7—Ag21	3.49	Ag31—$Ag24^{i}$	3.04	$Ag15^{i}$—$Ag17^{i}$	3.02
Ag8—Ag9	3.12	Ag31—$Ag12^{i}$	3.48	$Ag17^{i}$—$Ag43^{i}$	3.07
Ag8—Ag10	3.09	Ag32—Ag33	2.96	$Ag17^{i}$—$Ag31^{i}$	3.36
Ag8—$Ag33^{i}$	3.56	Ag32—Ag34	3.03	$Ag17^{i}$—$Ag32^{i}$	4.03
Ag9—Ag12	3.65	Ag32—$Ag9^{i}$	3.41	$Ag19^{i}$—$Ag21^{i}$	3.06
Ag9—$Ag32^{i}$	3.41	Ag33—Ag38	3.52	$Ag19^{i}$—$Ag43^{i}$	3.44
Ag9—Ag10	3.06	Ag33—$Ag8^{i}$	3.56	$Ag21^{i}$—$Ag22^{i}$	3.35
Ag10—Ag11	3.44	Ag33—Ag34	3.24	$Ag22^{i}$—$Ag28^{i}$	3.37
Ag11—Ag12	3.11	Ag34—Ag37	3.30	$Ag22^{i}$—$Ag26^{i}$	3.12
Ag11—Ag22	3.67	Ag34—Ag43	3.69	$Ag23^{i}$—$Ag25^{i}$	3.22
Ag11—Ag23	3.07	Ag37—Ag38	3.10	$Ag23^{i}$—$Ag26^{i}$	3.88
Ag12—Ag23	3.11	Ag37—Ag39	3.08	$Ag24^{i}$—$Ag25^{i}$	3.06
Ag12—$Ag31^{i}$	3.48	Ag38—Ag39	3.06	$Ag25^{i}$—$Ag40^{i}$	3.55
Ag14—Ag15	3.59	Ag38—$Ag2^{i}$	3.37	$Ag26^{i}$—$Ag28^{i}$	3.05
Ag14—Ag19	3.03	Ag39—Ag40	3.55	$Ag26^{i}$—$Ag39^{i}$	3.43
Ag14—Ag21	3.09	Ag40—$Ag1^{i}$	3.05	$Ag28^{i}$—$Ag19^{i}$	3.47
Ag15—Ag17	3.02	Ag40—$Ag2^{i}$	3.12	$Ag28^{i}$—$Ag37^{i}$	3.57
Ag15—Ag43	3.14	$Ag1^{i}$—$Ag2^{i}$	3.06	$Ag32^{i}$—$Ag33^{i}$	2.96
Ag15—$Ag24^{i}$	3.66	$Ag1^{i}$—$Ag6^{i}$	3.99	$Ag32^{i}$—$Ag34^{i}$	3.03
Ag17—Ag31	3.36	$Ag2^{i}$—$Ag5^{i}$	3.83	$Ag33^{i}$—$Ag34^{i}$	3.24
Ag17—Ag43	3.07	$Ag5^{i}$—$Ag6^{i}$	3.08	$Ag33^{i}$—$Ag38^{i}$	3.52
Ag17—Ag32	4.03	$Ag5^{i}$—$Ag7^{i}$	3.19	$Ag34^{i}$—$Ag37^{i}$	3.30
Ag19—Ag43	3.44	$Ag5^{i}$—$Ag8^{i}$	3.30	$Ag34^{i}$—$Ag43^{i}$	3.69
Ag19—Ag28	3.47	$Ag6^{i}$—$Ag7^{i}$	3.03	$Ag37^{i}$—$Ag39^{i}$	3.08
Ag19—Ag21	3.06	$Ag6^{i}$—$Ag14^{i}$	3.37	$Ag37^{i}$—$Ag38^{i}$	3.10
Ag21—Ag22	3.35	$Ag7^{i}$—$Ag10^{i}$	3.57	$Ag38^{i}$—$Ag39^{i}$	3.06
Ag22—Ag26	3.12	$Ag7^{i}$—$Ag21^{i}$	3.49	$Ag39^{i}$—$Ag40^{i}$	3.55

**Ag-Ag edge lengths in Ag_{24} shell of SD/Ag90a (Å)**					
Ag3—Ag4	3.09	$Ag36^{i}$—$Ag45^{i}$	3.02	Ag36—Ag35	3.47
Ag3—$Ag36^{i}$	3.14	$Ag36^{i}$—$Ag35^{i}$	3.47	$Ag35^{i}$—$Ag44^{i}$	3.14
Ag4—$Ag45^{i}$	3.19	$Ag45^{i}$—Ag13	3.46	$Ag35^{i}$—$Ag42^{i}$	3.06
Ag13—$Ag16^{i}$	3.09	$Ag16^{i}$—$Ag44^{i}$	3.43	$Ag44^{i}$—$Ag18^{i}$	3.05
Ag13—Ag27	3.08	Ag27—Ag42	3.48	$Ag27^{i}$—$Ag42^{i}$	3.48
Ag16—$Ag13^{i}$	3.09	Ag16—Ag44	3.43	$Ag18^{i}$—$Ag42^{i}$	3.09
Ag16—$Ag41^{i}$	3.17	$Ag13^{i}$—Ag45	3.46	$Ag3^{i}$—Ag36	3.14
Ag18—Ag42	3.09	$Ag13^{i}$—$Ag27^{i}$	3.08	Ag36—Ag45	3.02
Ag18—Ag44	3.05	$Ag41^{i}$—$Ag27^{i}$	3.12	$Ag16^{i}$—Ag41	3.17
Ag27—Ag41	3.12	Ag41—$Ag3^{i}$	3.44	$Ag4^{i}$—Ag45	3.19
Ag42—Ag35	3.06	$Ag3^{i}$—$Ag4^{i}$	3.09	Ag3—$Ag41^{i}$	3.44
Ag44—Ag35	3.14	$Ag4^{i}$—$Ag18^{i}$	3.43	Ag4—Ag18	3.43
**Ag-Ag edge lengths in Ag_6 shell of SD/Ag90a (Å)**					
Ag20—Ag29	3.58	Ag29—$Ag30^{i}$	3.56	Ag30—$Ag20^{i}$	3.51
Ag20—$Ag30^{i}$	3.51	Ag29—Ag30	3.61	$Ag20^{i}$—$Ag30^{i}$	3.61
Ag20—$Ag29^{i}$	3.60	Ag29—$Ag20^{i}$	3.60	$Ag29^{i}$—$Ag20^{i}$	3.58
Ag29—Ag30	3.59	Ag30—$Ag29^{i}$	3.56	$Ag29^{i}$—$Ag30^{i}$	3.59
Symmetry code: (i) $-x+1$, $-y+1$, $-z+1$.					

Supplementary Table 4: Axis alignment of icosahedral, octahedral and tetrahedral shells

Arrange-ment	Polyhedron	Vertices	Shell sym-metry order	Shell point group	Type (n-fold) and number of axes of rotational symmetry				Nest sym-metry order	Nest point group
					5-	4-	3-	2-		
	Dodecahedron	555	120	I_h	6		10	15		
	Icosahedron	33333	120	I_h	6		10	15		
	Cube	444	48	O_h		3	4	6		
	Octahedron	3333	48	O_h		3	4	6		
	Tetrahedron	333	24	T_h			4	3		
Kepler's Kosmos	Dodecahedron	555	120	I_h			4	3		
	Icosahedron	33333	120	I_h			4	3		
	Cube	444	48	O_h		3	4			
	Octahedron	3333	48	O_h		3	4			
	Tetrahedron	333	24	T_d			4	3		
	Quintuple nest						**4**	**3**	**24**	**T_d**
Kepler's Kosmos	Dodecahedron	555	120	I_h			4	3		
	Cube	444	48	O_h		3	4			
	Double nest						**4**	**3**	**24**	**T_h**
Alternative	Dodecahedron	555	120	I_h			4	3		
	Icosahedron	33333	120	I_h			4	3		
	Cube, 45°	444	48	O_h		3	4			
	Octahedron, 45°	3333	48	O_h		3	4			
	Tetrahedron	333	24	T_d			4	3		
	Quintuple nest							**3**	**4**	**D_2**
Alternative	Dodecahedron	555	120	I_h			4	3		
	Cube, 45°	444	48	O_h		3	4			
	Double nest							**3**	**8**	**D_{2h}**
**SD/Ag90a**	Rhombicosi-dodecahedron	3454	120	I_h			4	3		
	Truncated octahedron	466	48	O_h		3	4			
	Octahedron	3333	48	O_h		3	4			
	Triple nest						**4**	**3**	**24**	**T_h**

Supplementary References

1) Li, G., Lei, Z. & Wang, Q.-M. Luminescent molecular Ag-S nanocluster $[Ag_{62}S_{13}(SBu^t)_{32}](BF_4)_4$. *J. Am. Chem. Soc.* **132**, 17678-17679, (2010).

2) Rigaku Oxford Diffraction. *CrysAlis^{Pro} Software system, version 1.171.40.25a*, Rigaku Corporation: Oxford, UK, (2018).

3) Palatinus, L. & Chapuis, G. SUPERFLIP - a computer program for the solution of crystal structures by charge flipping in arbitrary dimensions. *J. Appl. Crystallogr.* **40**, 786-790 (2007).

4) Sheldrick, G.M. Crystal structure refinement with SHELXL. *Acta Crystallogr., Sect. C: Struct. Chem.* **71**, 3-8 (2015).

5) Dolomanov, O.V., Bourhis, L.J., Gildea, R.J., Howard, J.A.K. & Puschmann, H. OLEX2: a complete structure solution, refinement and analysis program. *J. Appl. Crystallogr.* **42**, 339-341 (2009).

6) Spek, A.L. Structure validation in chemical crystallography. *Acta Crystallogr D* **65**, 148-155 (2009).

7) Voss, N.R. & Gerstein, M. 3V: cavity, channel and cleft volume calculator and extractor. *Nucleic Acids Res* **38**, W555-W562 (2010).
