# Supplementary material for: A Keplerian Ag90 nest of Platonic and Archimedean polyhedra in different symmetry groups
Source: Nat Commun. 2020 Jul 3;11:3316. doi: 10.1038/s41467-020-17198-1 (PMC7335041; doi:10.1038/s41467-020-17198-1)
Supplement: Supplementary file 3 — Description of Additional Supplementary Files [file 41467_2020_17198_MOESM3_ESM.pdf]

Description of Additional Supplementary Files

File Name: Supplementary Movie 1
Description: Switching between Kepler's Kosmos and the Alternative alignment, showing the cube and the octahedron in the latter at 45º to their positions in the former. The viewer may stop the action at any point and switch between structures.
